# A pilot systematic genomic comparison of recurrence risks of hepatitis B virus-associated hepatocellular carcinoma with low- and high-degree liver fibrosis

**DOI:** 10.1186/s12916-017-0973-7

**Published:** 2017-12-07

**Authors:** Seungyeul Yoo, Wenhui Wang, Qin Wang, M Isabel Fiel, Eunjee Lee, Spiros P. Hiotis, Jun Zhu

**Affiliations:** 1 Department of Genetics and Genomic Sciences, Icahn Institute of Genomics and Multiscale Biology, Icahn School of Medicine at Mount Sinai, New York, NY USA; 2 Icahn Institute for Genomics and Multiscale Biology, Icahn School of Medicine at Mount Sinai, New York, NY USA; 3 Department of Surgery, Division of Surgical Oncology, Icahn School of Medicine at Mount Sinai, New York, NY USA; 4 Department of Pathology, Icahn School of Medicine at Mount Sinai, New York, NY USA; 5 Sema4, a Mount Sinai venture, Stamford, CT USA

**Keywords:** HBV-HCC, HBV integration, Fusion transcript, Pathogenic SNPs, Tumor recurrence, Liver fibrosis

## Abstract

**Background:**

Chronic hepatitis B virus (HBV) infection leads to liver fibrosis, which is a major risk factor in hepatocellular carcinoma (HCC) and an independent risk factor of recurrence after HCC tumor resection. The HBV genome can be inserted into the human genome, and chronic inflammation may trigger somatic mutations. However, how HBV integration and other genomic changes contribute to the risk of tumor recurrence with regards to the different degree of liver fibrosis is not clearly understood.

**Methods:**

We sequenced mRNAs of 21 pairs of tumor and distant non-neoplastic liver tissues of HBV-HCC patients and performed comprehensive genomic analyses of our RNAseq data and public available HBV-HCC sequencing data.

**Results:**

We developed a robust pipeline for sensitively identifying HBV integration sites based on sequencing data. Simulations showed that our method outperformed existing methods. Applying it to our data, 374 and 106 HBV host genes were identified in non-neoplastic liver and tumor tissues, respectively. When applying it to other RNA sequencing datasets, consistently more HBV integrations were identified in non-neoplastic liver than in tumor tissues. HBV host genes identified in non-neoplastic liver samples significantly overlapped with known tumor suppressor genes. More significant enrichment of tumor suppressor genes was observed among HBV host genes identified from patients with tumor recurrence, indicating the potential risk of tumor recurrence driven by HBV integration in non-neoplastic liver tissues. We also compared SNPs of each sample with SNPs in a cancer census database and inferred samples’ pathogenic SNP loads. Pathogenic SNP loads in non-neoplastic liver tissues were consistently higher than those in normal liver tissues. Additionally, HBV host genes identified in non-neoplastic liver tissues significantly overlapped with pathogenic somatic mutations, suggesting that HBV integration and somatic mutations targeting the same set of genes are important to tumorigenesis. HBV integrations and pathogenic mutations showed distinct patterns between low and high liver fibrosis patients with regards to tumor recurrence.

**Conclusions:**

The results suggest that HBV integrations and pathogenic SNPs in non-neoplastic tissues are important for tumorigenesis and different recurrence risk models are needed for patients with low and high degrees of liver fibrosis.

**Electronic supplementary material:**

The online version of this article (doi:10.1186/s12916-017-0973-7) contains supplementary material, which is available to authorized users.

## Background

Chronic infection with hepatitis B virus (HBV) is one of the primary risk factors for development of hepatocellular carcinoma (HCC). Viral proteins, such as HBx and truncated pre-S protein, have oncogenic properties by influencing diverse signaling pathways and changing expression level of host genes [1–4]. In addition, chronic HBV infection induces inflammation, oxidative stress, and a prolonged fibrotic response [5, 6]. This inflammatory and regenerative environment may lead to hepatocyte transformation and HCC development [7].

Integration of HBV DNA into the host genome contributes to hepatocarcinogenesis by inducing genomic instability and altering expression of cancer-related genes [8–11]. With the advances of genome-wide sequencing techniques, it is possible to identify HBV DNA integration sites in the human genome [12]. Sung et al. [13] studied HBV integration in 81 HCC patients using Whole Genome Sequencing (WGS) and reported 344 and 55 HBV integration events in tumor and normal liver tissues, respectively. Jiang et al. [14] reported 255 HBV integration sites from WGS profiles of four HCC patients and found more integration sites in tumor tissues. Ding et al. [15] devised a massive anchored parallel sequencing to isolate and sequence HBV integrants of 40 pairs of HCC and normal tissues and identified 296 HBV integration events; while they detected a similar set of host genes as other studies, they reported fewer integration events in tumors compared to normal tissues. Chiu et al. [16] studied HBV fusion transcripts of 16 pairs of HBV–HCC and their corresponding normal tissues and found 413 and 94 unique integration sites from normal and tumor tissues, respectively. In these studies, HBV integration events were observed in a few common host genes, including *KMT2B* (also known as *MLL4*), *FN1*, and *TERT*, while integration events in many other host genes were unique to each study. This suggests that HBV integration might be a random event associated to physical properties [14] across the whole genome; however, it is not yet clear how HBV integration events are associated with the disease phenotypes and progression. Most existing studies have identified and characterized HBV integration events at the DNA level, yet whether HBV integration into the human genome impacts gene function or expression remains to be fully characterized.

HCC is notorious for the high risk of tumor recurrence even after successful surgical resection [17]. HCC recurrence is closely associated with overall survival (Additional file 1: Figure S1). Patients with a high risk for recurrence may be considered for adjuvant therapies or liver transplant without liver resection – it has been shown that survival after liver transplant dramatically decreased for patients having previously undergone liver resection [18]. Unlike hepatitis C-associated HCC, where the majority of cancers form through orderly progression from chronic inflammation, fibrotic injury and liver cirrhosis, HBV-associated HCC can develop in livers of varying degrees of fibrosis [19, 20]. In our previous study based on a cohort of 189 HBV-HCC patients in New York City, 35% of HBV-HCC developed in livers with low fibrosis (histologically defined as Ishak stage 0–3) [20]. HCC patients with minimal liver fibrosis remain a poorly defined subgroup and the molecular mechanisms underlying hepatocarcinogenesis are not yet clear understood since most of the previous genomic studies of HBV-HCC have focused on patients with cirrhosis. Whether the same genetic and genomic features lead to hepatocarcinogenesis and HCC recurrence after tumor resection in HCC patients with low fibrosis or cirrhosis is not well studied.

**Fig. 1:**
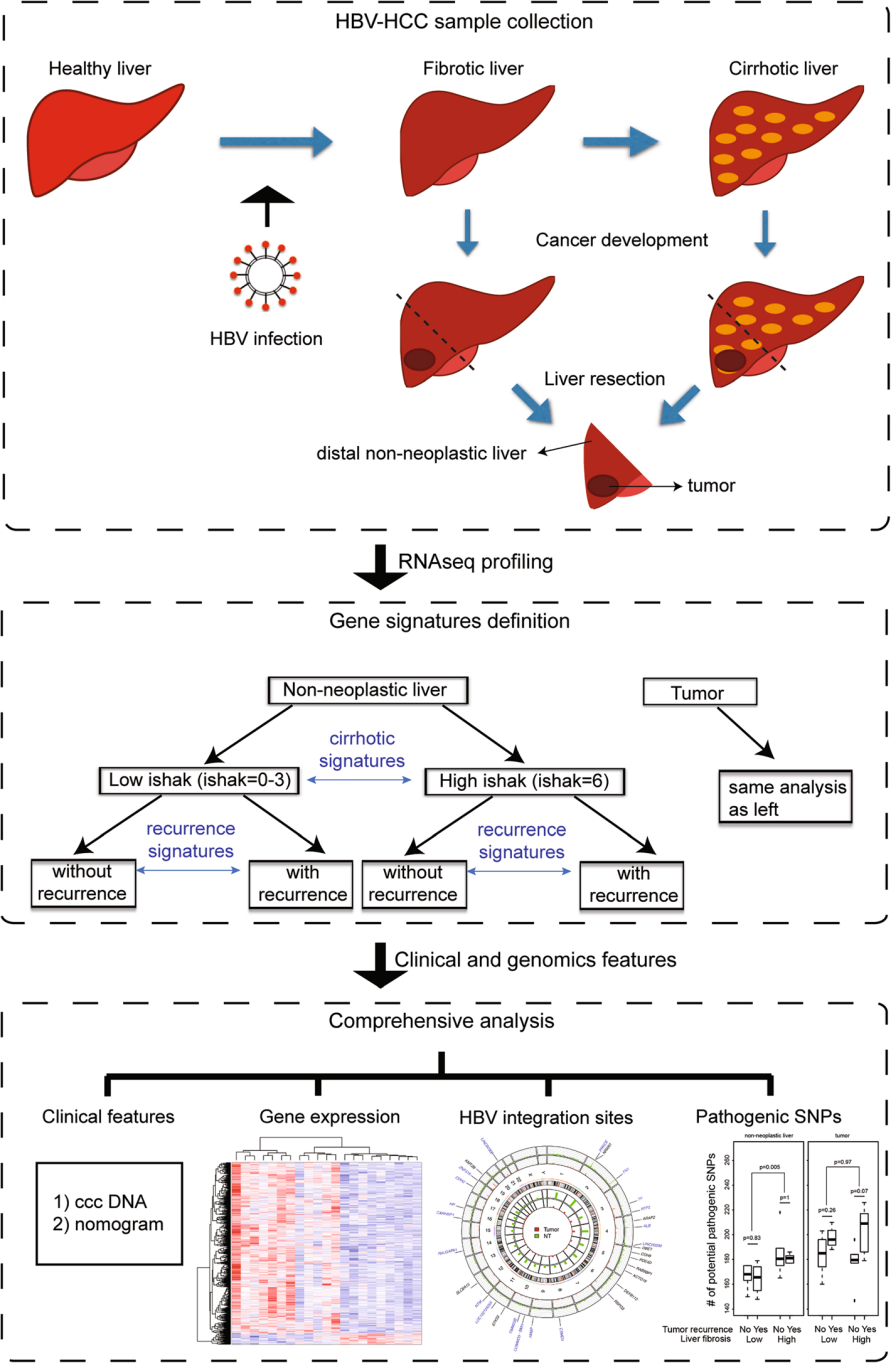
Study overview – assessment of differences in HBV-HCC tumor recurrence in patients of low and high liver fibrosis stage. Twenty-one pairs of non-neoplastic liver and HBV-HCC tumor samples of varying liver fibrosis status were collected from surgical resection and their transcriptome was profiled through RNAseq technique. Their clinical and genomic features were compared through comprehensive analysis based on liver fibrosis stage and tumor recurrence status

Herein, we focus on a comparison of genomic features associated with high risk for HCC recurrence in HCC patients with low or end-stage fibrosis (Fig. 1). We aim to assess the impact of clinical parameters (liver fibrosis, tumor size and differentiation), HBV integration, and other genomic features on the risk of HCC recurrence. We performed transcriptome profiling in paired tumor and distant non-neoplastic liver tissues of 21 patients with minimal fibrosis or end-stage fibrosis (Methods) using paired-end sequencing technology. First, we applied a systematic approach to identify the viral-human gene fusion transcripts in both tumor and non-neoplastic liver tissues of the 21 patients. We developed a robust pipeline modified from VirusFinder [21] to identify HBV integration sites in tumor and non-neoplastic liver tissues. HBV integration events and human transcripts with HBV integration were characterized in tumor and non-neoplastic liver tissue. Unlike previous studies, we focused on HBV-human gene fusion transcripts, which represent a small fraction of HBV integration events but likely result in biological changes in host cells. Next, we compared potential pathological SNP loads in mRNA transcripts with regard to HCC recurrence and stage of liver fibrosis. Our observations suggest that there are different genomic features and tumorigenesis mechanisms associated with the risk of HBV-HCC recurrence in patients with different liver fibrosis stages. Although our sample size is small and further validation is required, some of our observations replicated previous HBV-HCC sequencing studies.

## Methods

### Patients, histopathologic assessment, and follow-up

For this RNA sequencing study, a total of 21 pairs of tumor and non-neoplastic liver tissue samples were selected from HBV-HCC patients who underwent primary surgical resection at the Mount Sinai Medical Center in New York, NY, USA, between 2008 and 2013. Prior to study initiation, all aspects of the research were approved by the Icahn School of Medicine Institutional Review Board. The study protocol conformed to the ethical guidelines of the 1975 Declaration of Helsinki.

The cohort of this RNAseq study is a subset of a cohort previously described [22]. Patients were assessed pre-operatively by dynamic axial imaging (three-phase computerized tomography with intravenous contrast or multi-phase magnetic resonance imaging with intravenous contrast). Liver resection was performed in patients with surgically resectable disease and well-preserved synthetic liver function as assessed by normal serum total bilirubin, albumin, and international normalized ratio. Patients with portal hypertension as evidenced by a platelet count < 100 × 10^3^/μL, peri-esophageal or peri-splenic varices on axial imaging, or a portal-systemic venous pressure gradient ≥ 10 mm Hg were excluded from liver resection. This cohort included only Child–Pugh A cirrhotic patients since patients with clinical evidence of Child-Pugh B–C cirrhosis were generally not suitable for liver resection surgery.

This RNAseq pilot study included patients who (1) had the largest tumor diameter smaller than 5 cm; (2) had either minimal liver fibrosis (Ishak stage 0–3) or end-stage liver fibrosis (Ishak stage 6) as determined by dedicated pathology review by a single liver pathologist [20]; and had (3) paired fresh frozen tumor and non-neoplastic liver tissue as well as (4) intrahepatic HBV viral DNA copy numbers available. Median follow-up of the survivors was 49 months (4–90 months). There were more males than females included in the study, which is consistent with the sex bias in HBV-HCC [23]. A summary of the clinical information of patients in this study is listed in Table 1. Note that no patient underwent liver transplantation prior to HCC recurrence. One patient, P16, had liver transplantation after HCC recurrence.

**Table 1:** Summary of clinical information of the 21 patients included in the Mount Sinai dataset

Clinical characteristics	Group			
	Low fibrosis		End-stage fibrosis	
	Recurrent	Non-recurrent	Recurrent	Non-recurrent
Number of patients	4	6	5	6
Age, years (mean ± SD)	51 ± 16.8	51.2 ± 10.2	54.6 ± 11.1	55.3 ± 7.4
Sex (M/F)	4/0	3/3	5/0	2/4
Follow-up, months (mean ± SD)	31.5 ± 1.3	56.2 ± 7.3	16.6 ± 8	65.2 ± 16.4

### Transcriptome profiling using RNAseq

All tissue samples used for RNAseq were collected from the first surgical resection. Total RNAs (1–3 μg/sample) extracted from surgical resection specimens were submitted to the Mount Sinai Genomic Core Facility for quality control analysis. The RNA quality was assessed using the Agilent 2100 Bioanalyzer, and the RNA integrity numbers for all 21 pairs of samples were approximately 8.2 ± 0.7 (mean ± SD). The poly(A)-RNA was captured using oligo-dT beads and used for cDNA library preparation using the standard TruSeq RNA Sample Prep Kit v2 protocol (Illumina, CA, USA). Briefly, total RNA was poly(A)-selected and then fragmented. The cDNA was synthesized using random hexamers, end-repaired and ligated with appropriate adaptors for sequencing. The library then underwent size selection and purification using AMPure XP beads (Beckman Coulter, CA, USA). The appropriate Illumina-recommended 6-bp barcode bases were introduced at one end of the adaptors during the PCR amplification step. The size and concentration of the RNAseq library was measured by Bioanalyzer and Oubit fluorometry (Life Technologies, NY, USA) before loading onto the sequencer. The mRNA libraries were sequenced on the Illumina HiSeq 2500 System with 100 nucleotide paired-end reads, according to the standard manufacturer’s protocol (Illumina, CA, USA). Sequence reads were aligned to human transcript reference sequences from the ENSEMBLE database (Homo_sapiens.GRCh37.55.cdna.all.fa) for the expression analysis at gene/transcript levels using TopHat and HTSeq softwares [24, 25]. The raw fastq sequences and the normalized RPKM matrix were deposited in Gene Expression Omnibus database with accession number GSE94660. The HBV reference genome sequence, NC_003977.1, was downloaded from the NCBI database (https://www.ncbi.nlm.nih.gov/nuccore/NC_003977.1) to map reads onto viral transcripts.

### Validation sets for HBV integration

DNAseq [13] and RNAseq [26] data for nine paired HCC tumor and adjacent normal tissue samples in a BGI HCC study are publicly available. The WGS data were downloaded from the European Genome-phenome Archive under accession number ERP001196. RNAseq data were downloaded from NCBI Sequence Read Achieve under accession number SRA074279. We ran our pipeline on the DNA sequencing data of 11 N, 11 T, 22 N, 22 T, 30 N, 30 T, 70 N, 70 T, 82 N, 82 T, 180 N, 180 T, 200 N, and 200 T. At the same time, we ran our pipeline on RNAseq data of 18 samples separately (28 N, 28 T, 65 N and 65 T in extra). The integration sites detected from DNAseq and RNAseq data, as well as experimentally validated ones, were used to validate our pipeline and results. In addition, we downloaded RNAseq data of 21 pairs of HBV-positive HCC tumors and corresponding non-tumor tissues in the TCGA Liver Hepatocellular Carcinoma (LIHC) dataset (https://gdc-portal.nci.nih.gov/legacy-archive/search/f). Among these patients 13, 5, and 2 were white, Asian, and African-American, respectively; the ethnicity of one patient was unknown. We also downloaded transcriptome sequencing data of 21 pairs of non-tumor and HBV-associated HCC [27] from the International Cancer Genome Consortium (ICGC, https://icgc.org). Detailed information of the TCGA and ICGC samples used in our study is shown in Additional file 2: Table S1. Additional RNAseq dataset from Chiu et al. [16] with 16 paired HCCs and non-tumorous livers (SRA ID: SRP062885) were also used for pathogenic SNP load analysis.

### A robust pipeline for identification of HBV integration sites

VirusFinder is an automated virus-host integration detection software package that can deal with virus-induced host genome instability and viral genome variability [21, 28]. It has been shown that VirusFinder performs better than other state-of-the-art virus integration detection pipelines such as VirusSeq [29] and VirusFusionSeq [30] in terms of both accuracy and time efficiency [28]. Our virus integration detection pipeline was based on VirusFinder, with several modifications. Firstly, more candidate sequences were analyzed through our pipeline. One of the main differences was the addition of a re-mapping and confirmation step after potential integration sites were identified to increase pipeline sensitivity and specificity in identifying HBV integration sites (detailed in Additional file 3: Supplementary Materials and Methods). Multiple and different simulation studies were performed to compare HBV identification accuracy between our pipeline and VirusFinder (Additional file 3: Supplementary Materials and Methods).

### Quantitative intrahepatic HBV DNA and cccDNA measurements

The procedure was described previously [22] (detailed in Additional file 3: Supplementary Methods). In brief, HBV DNA and cccDNA were amplified from genomic DNA extracted from surgically resected tumor or non-neoplastic liver specimens using the QIAamp DNA extraction kit (Qiagen) [31]. A spectrophotometric ratio of absorbance at 260 nm and 280 nm between 1.8 and 2.0 was assured in all genomic DNA samples. Quantitative PCR was standardized to the human albumin copy number in order to determine the viral DNA copy number/hepatocyte.

### Pathogenic SNP load

For each RNAseq sample, we inferred SNP variants based on RNAseq following the suggested workflow of GATK Best Practices (https://software.broadinstitute.org/gatk/documentation/article.php?id=3891). This workflow is designed specifically for SNP calling based on RNAseq data by modifying original workflow for DNAseq [32]. The workflow consists of the following steps: (1) mapping raw RNAseq reads to reference based on STAR 2-pass alignment [33]; (2) adding read groups, sorting, marking duplicates, and indexing through Picard processing steps; (3) splitting reads into exon segments and hard-clipping any sequences overhanging into the intron regions, (4) base recalibration, and (5) variant calling and filtering using GATK tools. Every parameter was set as default presented in the guide. After inferring the genotype of each sample, tumor variants were compared with those of matching non-neoplastic liver to define somatic mutations for individual SNPs and somatic mutations called for each patient were compared with potential pathogenic SNPs curated in COSMIC mutation data [34]. Pathogenic mutations were defined by Functional Analysis through Hidden Markov Models, which predict the functional consequences of sequence variants [35].

## Results

Most previous HBV-HCC studies have focused on cirrhosis patients as it is commonly believed that there is a linear path from chronic inflammation induced by HBV infection to liver cirrhosis, and subsequently to hepatocarcinogenesis [36]. However, 35% of HBV-HCC patients have low liver fibrosis [20]. Herein, we systematically compared the clinical and genomic features associated with HCC recurrence risk in patients with different liver fibrosis stages (Fig. 1).

### Clinical features for predicting HCC recurrence risk

**Fig. 2:**
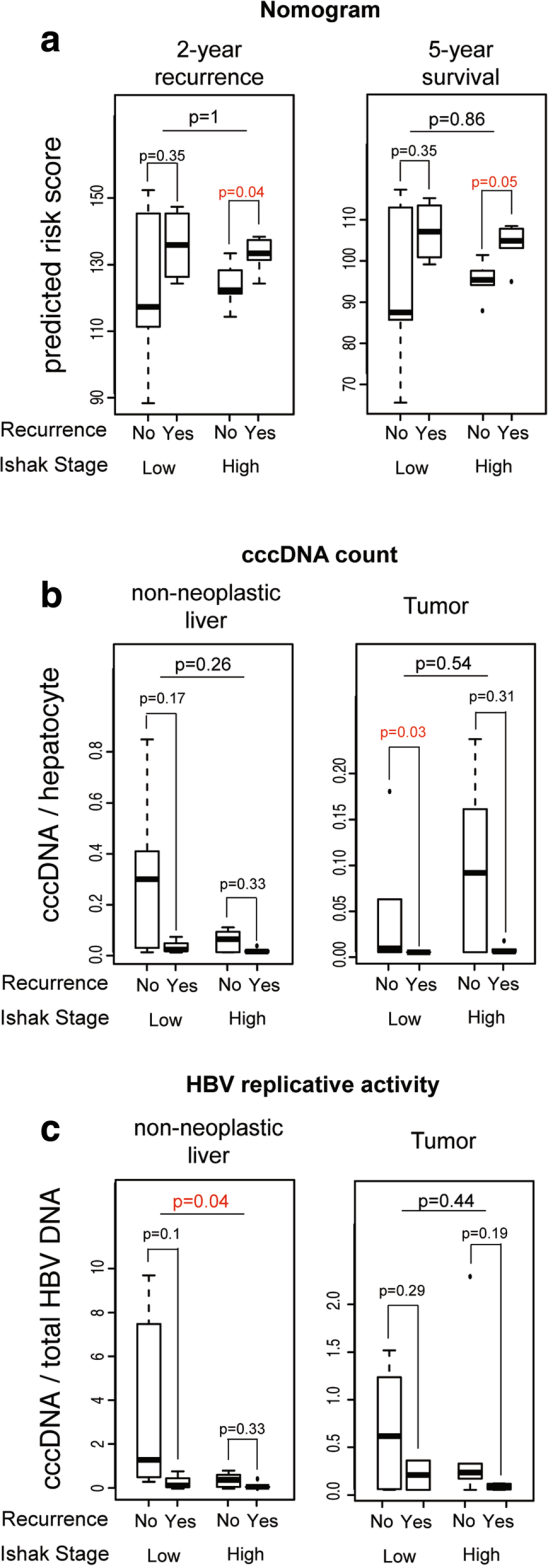
Association of clinical features with tumor recurrence in low and high liver fibrosis. **a** Predicted nomogram scores of risks for 2- or 5-year recurrence was compared. Both cccDNA per hepatocyte (**b**) and HBV replicative activity (**c**) were compared between groups with and without tumor recurrence in patients of different liver fibrosis stages in non-neoplastic liver and tumor tissues. Wilcoxon rank sum test *P* value was used to measure the significance of the difference. Significant associations (*P* < 0.05) with tumor recurrence were marked in red color

A prognostic nomogram based on clinicopathologic data was developed to predict 2- and 5-year recurrence-free survival [37]. The nomogram scores were calculated for the Mount Sinai dataset and compared between patients with or without cancer recurrence. Higher scores were observed in patients with cancer recurrence (Fig. 2a). However, the nomogram scores for recurrence after 2 or 5 years significantly correlated with the recurrence status only in patients with end-stage fibrosis (Ishak, 6), but not in those with low liver fibrosis (Ishak, 0–3). This result suggests that different recurrence risk models are needed for HCC patients in early or late stage of liver fibrosis and that there may be different underlying mechanisms of tumor recurrence between the two groups.

Our previous studies indicate that intrahepatic cccDNA count and HBV replicative activity were associated with overall survival [22, 31]. Herein, we compared cccDNA counts and HBV replicated activities with regards to HCC recurrence in low and high fibrosis groups (Fig. 2b, c). In general, cccDNA counts were lower and HBV replicative activities were higher in non-neoplastic liver tissues of HCC recurrence for both low and high fibrosis groups. However, the differences were not significant due to the small sample size. We next examined genomic features and underlying molecular mechanisms associated with tumor recurrence in patients with low and high stage of liver fibrosis.

### Gene expression was not associated with HBV-HCC recurrence

In our previous study, we reported a set of differentially expressed genes in non-neoplastic liver between low and high Ishak staged patients [22]. Herein, the fibrosis stage signatures consistently overlapped with liver cancer survival or recurrence signatures, respectively (detailed in Additional file 3: Supplementary Results, Additional file 4: Table S2), suggesting a prognostic value of fibrosis stage. No significant gene expression change was found between groups with and without recurrence in low or high liver fibrosis in both non-neoplastic liver and tumor tissues. Existing prognosis signatures, including prognostic signatures from Hoshida et al. [38], failed to classify our samples into tumor recurrent or non-recurrent groups (detailed in Additional file 3: Supplementary Results, Additional file 5: Figure S2). This is not surprising given that our samples were specific for HBV-associated HCC with various stages of liver fibrosis. These results indicate that we need to explore other genomic features (e.g., HBV integration sites and SNP patterns) associated with tumor recurrence risk in low or high liver fibrosis groups.

### HBV integration identification

**Fig. 3:**
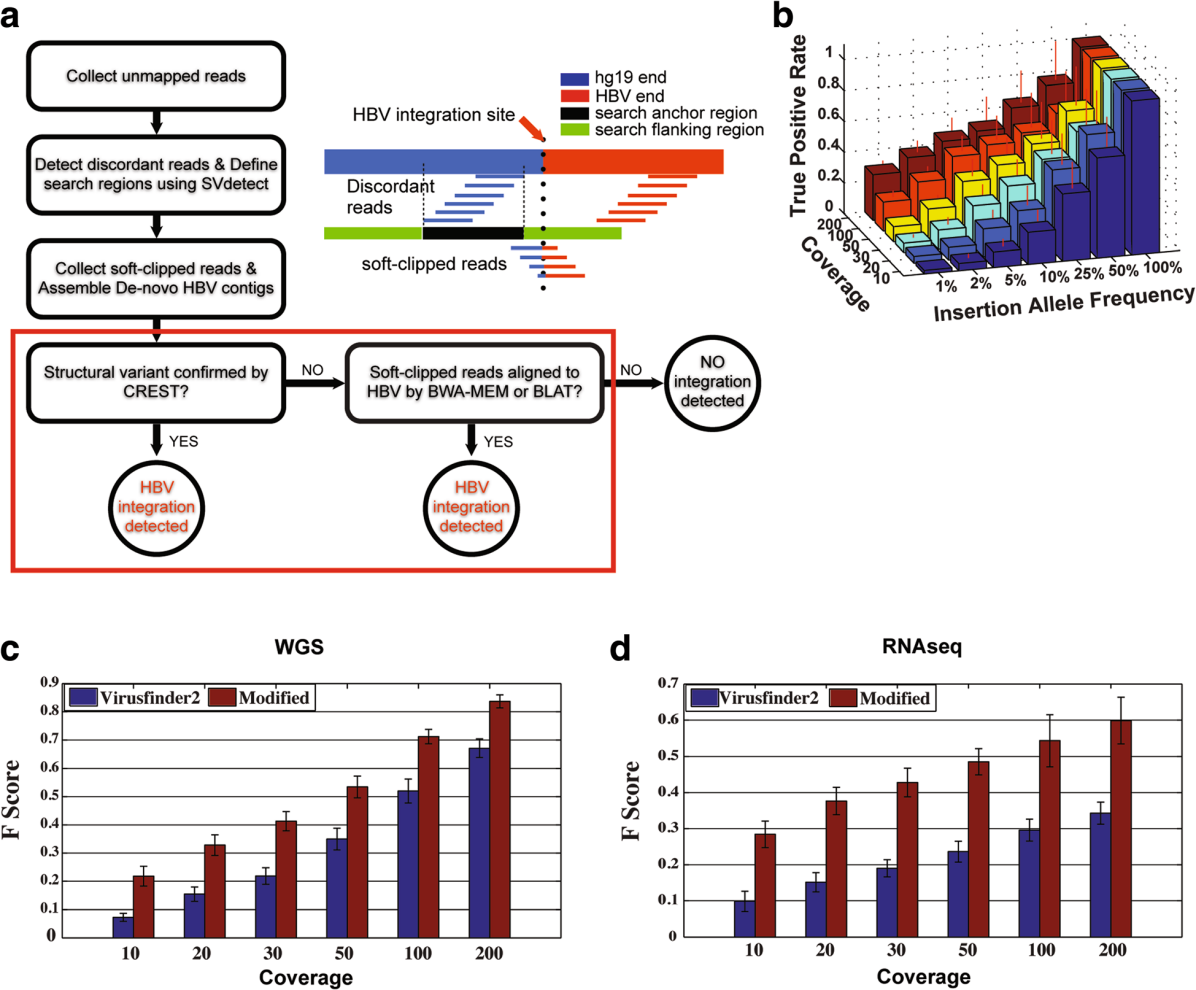
HBV integration identification. **a** The pipeline for HBV integration detection (detailed procedures for each step are described in Methods). The step indicated in the red box was the additional step in our pipeline. **b** The prediction accuracy (true positive rate) was evaluated through simulations with different HBV insertion allele frequency and coverage using simulated datasets. The performance of our pipeline was compared with results from Virusfinder2 across different coverage using simulated datasets of DNA (**c**) and RNA (**d**) sequencing. The detailed procedure of simulations is described in Additional file 3: Supplementary Materials and Methods

After HBV infection, HBV can insert its genome into the human genome and induce multiple hepatocarcinogenesis events. The power to identify a HBV insertion event depends on the HBV insertion allele frequency (IAF) and sequencing depth and coverage [39]. To enhance the power to detect insertion events of low IAF we modified VirusFinder [21] in multiple steps and developed our own pipeline for HBV integration site detection (Fig. 3a, Methods). Our simulation studies (described in Additional file 3: Supplementary Materials and Methods) suggested that a large fraction of integration sites were not detected at 10× coverage of whole genome sequencing (Fig. 3b). When VirusFinder and our pipeline were applied to the same simulated datasets, our pipeline resulted in more accurate predictions for integrations with low IAFs than VirusFinder in both DNA and RNA sequencing data (Fig. 3c, d). To further validate our pipeline, we applied it to a publically available HBV-HCC dataset, referred to as the BGI dataset, which consists of both whole genome sequencing [13] and RNA sequencing data [26] of the same patients (Methods). Based on WGS data, our pipeline identified 90% (9/10) and 81% (26/32) of the HBV integration sites reported by Sung et al. [13] in normal and tumor tissues, respectively; a few of the integration sites reported by Sung et al. (1 and 6 in normal and tumor tissues, respectively) but not detected by our pipeline were due to low alignment qualities and regions with unknown sequences (Additional file 6: Figure S3, Additional file 3: Supplementary Materials). When applied to RNAseq data in the BGI dataset, our pipeline identified more integration sites than those identified based on WGS data. Additionally, more integration sites in adjacent normal tissues were identified than in tumor tissues based on both WGS and RNAseq data (Additional file 7: Table S3). Interestingly, 24 and 2 integration sites were identified based on both WGS and RNAseq data by our pipeline, but not by Sung et al. [13], in normal and tumor tissue, respectively, suggesting that our approach is sensitive in detecting true HBV integration sites. This observation is consistent with our simulation results that the low sequence depth in WGS is disadvantageous for detecting integration sites, especially in normal tissue, where a relatively lower HBV IAF is expected compared to tumor tissues with clonal expansion [40]. It is also supported by the fact that, generally, more integration sites were obtained from RNAseq than WGS since RNAseq is typically focused on transcript regions with more than tens or hundreds of millions of reads [39]. We also compared HBV integration sites in the TCGA dataset by our pipeline and those from a recent TCGA paper [41], with the results suggesting that our pipeline had greater sensitivity and specificity (Additional file 8: Table S4, Additional file 3: Supplementary Materials).

### Characterize HBV integration sites identified in the Mount Sinai dataset

We applied our pipeline to the RNAseq data for the 21 pairs of non-neoplastic liver and tumor tissues from Mount Sinai (Methods). A total of 407 and 118 unique integration sites within 374 and 106 unique host genes with HBV integration were identified in normal and tumor tissues, respectively (Table 2). All of identified HBV integration sites for non-neoplastic liver and tumor tissues are listed in Additional file 9: Table S5. It is worth noting that the number of host transcripts with HBV S ORF integrated in both non-neoplastic liver and tumor tissues was significantly correlated with serum HBsAg levels (Additional file 10: Figure S4A). Further, the trends were similar for the number of all host transcripts with HBV integration (Additional file 10: Figure S4B), suggesting that fusion transcripts with HBV S ORF may partially contribute to HBsAg levels in serum.

**Table 2:** Summary of HBV integration events in Mount Sinai dataset

Sample	Ishak	Recurrent with 5 years	Months to recurrence or last follow-up	Nomogram		HBV integration host genes	
				2 year	5 year	Non-neoplastic liver	Tumor
P105	6	0	66	121	94.75642	20	9
P106	2	1	34	147	114.6344	0	0
P112	6	0	46	123	93.59073	6	4
P131	3	0	62	145	112.3616	30	4
P138	6	1	26	138	107.8455	5	1
P140	6	1	17	131	102.5062	7	0
P152	6	1	4	124	94.4346	6	3
P157	1	0	54	88	64.96545	34	21
P158	2	0	52	152	116.7212	4	1
P161	0	1	29	128	101.9273	0	1
P16	6	1	17	133	104.2455	11	9
P170	1	0	44	120	85.11677	60	5
P171	2	0	46	114	87.53396	10	2
P176	6	0	42	133	100.8071	43	12
P179	2	0	43	111	86.34	17	6
P49	6	0	58	121	94.91296	0	3
P62	6	0	73	114	87.33525	0	2
P6	6	0	78	128	97.04106	17	15
P75	2	1	32	143	111.0713	42	2
P94	6	1	18	137	107.1909	14	18
P99	2	1	31	124	98.59824	75	0

**Fig. 4:**
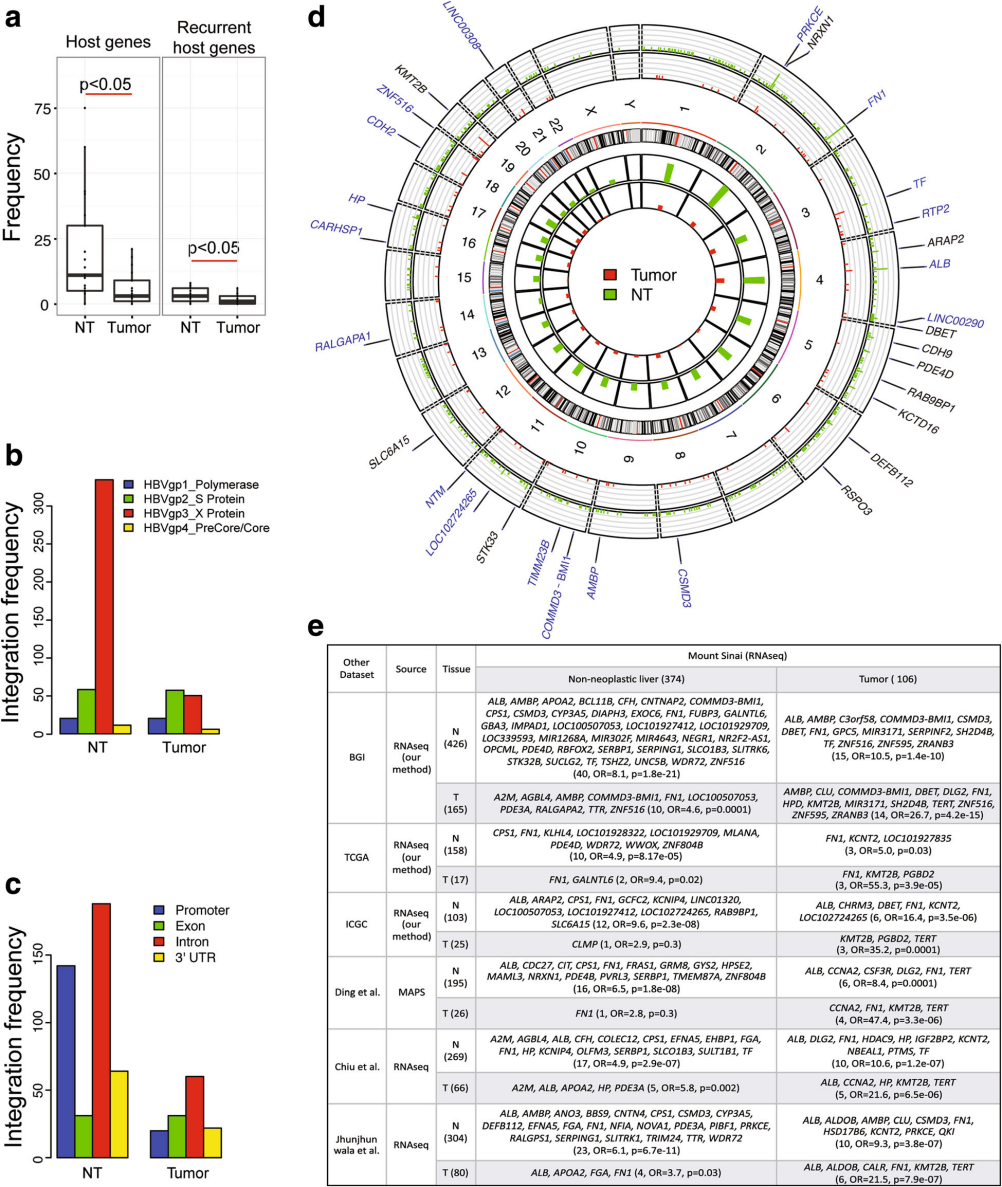
Characterization of HBV integration sites in Mount Sinai dataset. **a** The number of unique fusion transcripts and recurrent fusion transcripts was compared between non-neoplastic liver and tumor tissues. *P* value was measured from Wilcoxon rank sum test. **b**, **c** The distribution of HBV integration sites (407 in non-neoplastic liver and 118 in tumor tissues) in viral genome (**b**) and human transcripts (**c**). **d** Genome-wide distribution of HBV host genes (374 in normal and 106 in tumor) across entire chromosomes. Names of host genes are shown if they were observed from more than two samples. Labels in blue indicate when the host gene was identified both in normal and tumor. **e** Host transcripts with HBV integration in the Mount Sinai dataset are compared with results from other datasets. For BGI, TCGA, and ICGA datasets, HBV integration sites were identified from our pipeline. The significance of overlap was tested based on Fisher’s exact test

A more than three-fold HBV integration was observed in non-neoplastic tissue compared to tumor tissue, indicating that HBV integration patterns in non-neoplastic tissues are more diverse, consistent with recent results by Chiu et al. [16]. While most HBV fusion transcripts were detected only in one sample, 30 host transcripts with HBV fusion were detected in more than one sample (recurrent integration), and 18 of them were detected in both tumor and non-neoplastic liver tissue (Additional file 11: Table S6). A comparison of HBV integration in tumor versus paired non-neoplastic liver tissues showed a higher number of host transcripts with HBV integration and transcripts with recurrent HBV integration in non-neoplastic liver tissues (Wilcoxon test *P* = 0.002 and 0.03, respectively, as shown in Fig. 4a). Consistently, more host transcripts with HBV integration were identified in non-neoplastic liver tissues than in the paired tumor tissues when our pipeline was applied to BGI, TCGA, and ICGC HBV-HCC RNAseq datasets (Additional file 12: Figure S5).

To check whether preferential integration sites exist for HBV integration, the breakpoints of integration were counted in both the human and HBV genomes. HBV X gene transcript was more dominantly fused with human genome than other HBV transcripts, especially in normal samples (Fig. 4b), consistent with previous reports [11, 16]. More precisely, the breakpoint in the HBV genome preferentially occurred around nucleotides at nt1818 (Additional file 13: Figure S6A), consistent with previous reports [12, 13, 15]. In the human genome, HBV integration occurred mainly in the gene promoter and intron regions in non-neoplastic liver, while the intron region was the preferential integration site in tumor (Fig. 4c). Only 5–16% of all sequencing reads in each sample were mapped to intronic regions (Additional file 13: Figure S6B), consistent with ratios observed in other studies [42, 43]. However, HBV integrations preferentially occurred in promoter and intronic regions (Fig. 4c), suggesting regulatory roles of HBV integration in fusion gene expression. Moreover, Chiu et al. [16] reported that intronic HBV integrations have oncogenic properties. This pattern of HBV integrations preferentially occurring in gene promoter and intronic regions was also identified in the BGI and TCGA LIHC datasets (Additional file 13: Figure S6C), which was consistent with previously reported studies based on transcriptome sequencing [12, 16]. HBV integration sites were observed across entire chromosomes, while chromosome 1, 2 and 4 contained more than 30 fusion transcripts in non-neoplastic liver tissues (Fig. 4d).

HBV fusion transcripts identified in the Mount Sinai dataset were compared with integration results identified in other datasets or reported in previous studies [12, 15, 16] (Fig. 4e). Our results significantly overlapped with the HBV host transcripts identified based on RNAseq data of BGI (Fisher’s exact test (FET) *P* =1.8 × 10^-21^ and 4.2 × 10^-15^ for non-neoplastic liver and tumor tissues, respectively), TCGA LIHC dataset (FET *P* = 8.2 × 10^-5^ and 3.9 × 10^-5^ for non-neoplastic liver and tumor tissues, respectively), and ICGC HBV-HCC RNAseq dataset (FET *P* = 2.3 × 10^-8^ and 0.0001 for non-neoplastic liver and tumor tissues, respectively). Individual HBV integration sites identified in these dataset are listed in Additional file 14: Table S7 and were also consistent with previously reported HBV fusion transcripts in several previous studies (Fig. 4e) [12, 15, 16]. While some fusion transcripts were commonly found in both tumor and normal tissues across different datasets, several HBV fusion transcripts were restricted to normal or tumor tissues. For example, some known oncogenes, such as *KMT2B* and *TERT*, were dominantly observed in tumor while fusion transcripts with *CYP3A5*, *SERPING1*, and *WDR72* were only found in normal tissue. The most frequently identified fusion transcript in our dataset was *FN1* (8/42, 19%); however, the frequency was biased towards normal samples (7 and 1 occurrence in normal and tumor tissues, respectively). This was consistent with previous studies indicating that *FN1* is frequently targeted for HBV integration at the transcript level [44], but that it is not a cancer driver gene.

**Fig. 5:**
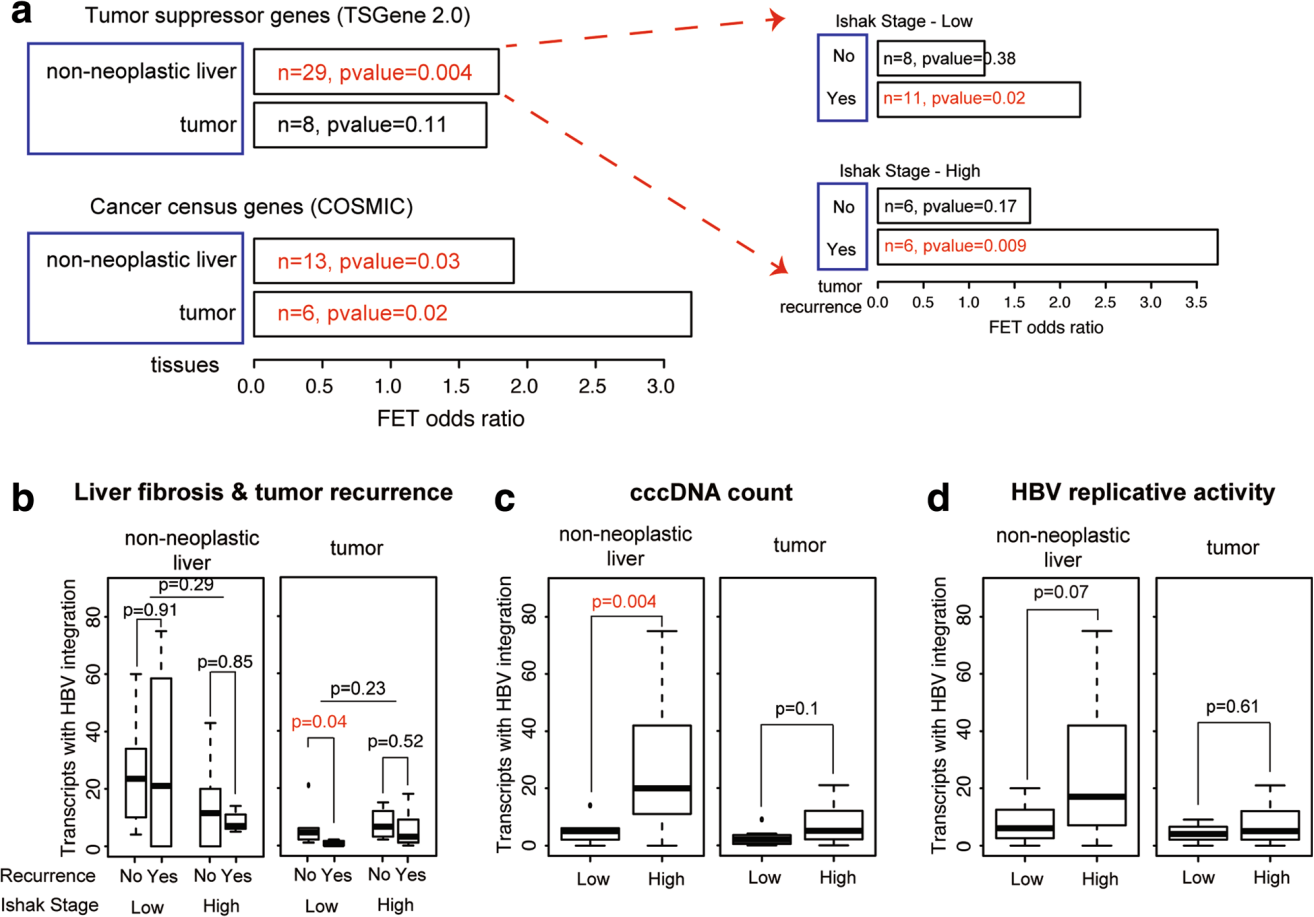
Association of HBV integration events and tumor recurrence. **a** Host genes with HBV integration events are significantly enriched for tumor suppressor genes [45] and cancer census genes [46]. In particular, only fusion transcripts identified in non-neoplastic tissues of patients with recurrence were enriched for tumor suppressor genes. “n” is the number of overlapped genes with tumor suppressor genes and p is Fisher’s exact test *P* value. **b** Association of the number of fusion transcripts and tumor recurrence in non-neoplastic and tumor tissues of low and high liver fibrosis. **c** Association of the number of fusion transcripts and cccDNA per hepatocyte or **d** HBV replicative activity within non-neoplastic liver and tumor tissues.

Host genes with HBV integration in non-neoplastic liver tissues were enriched for biological processes such as cell adhesion (*P* = 0.0002) and Wnt receptor signaling pathway (*P* = 0.005), whereas those in tumor tissues were enriched for platelet degranulation and activation (*P* = 4.9 × 10^-5^) (Additional file 15: Table S8). Detailed results of functional analysis for the host genes with HBV integration are reported in Additional file 3: Supplementary Materials and Methods. Host genes with HBV integration detected in non-neoplastic tissues were significantly enriched for tumor suppressor genes [45] (*P* = 0.004; Fig. 5a, Additional file 16: Table S9). In addition, the host genes with HBV integration significantly overlapped with COSMIC cancer census genes [46] (*P* = 0.03 and 0.02 for non-neoplastic and tumor tissues, respectively), suggesting that cells with these HBV integrations likely resulted in a growth advantage during clonal expansion. HBV-human gene fusion events may alter the host gene expression (Additional file 3: Supplementary Materials and Methods). For example, *KMT2B* expression level was higher in tumor tissues in which HBV-KMT2B fusion transcripts were detected (Additional file 17: Figure S7A).

### HBV integration and HCC tumor recurrence

Host transcripts with HBV integration identified in non-neoplastic liver tissues in HCC recurrence groups were significantly enriched for tumor suppressor genes [45], while those in non-recurrence groups were not (Fig. 5a, Additional file 18: Table S10). The number of host transcripts with HBV insertion identified in non-neoplastic liver tissues in recurrence groups was less than that identified in non-recurrence groups for both low and high fibrosis (Fig. 5b, left), but the differences were not significant. In conjunction, these results suggest that there are selective clonal expansions in non-neoplastic liver tissues with a high risk for HCC recurrence.

Similarly, the number of host transcripts with HBV integration identified in tumor tissues in recurrence groups was lower than that identified in non-recurrence groups for both low and high fibrosis (Fig. 5b, right), and the difference in the low fibrosis group being statistically significant (*P* = 0.04). This further suggests that the tumorigenesis mechanisms for low and high fibrosis groups are likely different and therefore the exact tumorigenesis mechanism for each group needs further investigation.

### Number of HBV integration sites associated with HBV cccDNA counts

To investigate what factors determine the number of host transcripts with HBV integration we compared these with HBV cccDNA count and HBV replicative activity (Additional file 2: Table S1). The larger number of HBV integration events was significantly associated with higher HBV cccDNA counts in non-neoplastic liver tissues (Wilcox test *P* = 0.004, Fig. 5c); this was also the trend in tumor tissues. There was a similar pattern between the number of HBV integration events and HBV replicative activity, but the association was not statistically significant (Fig. 5d).

### Pathogenic SNP loads and HBV-HCC tumor recurrence

**Fig. 6:**
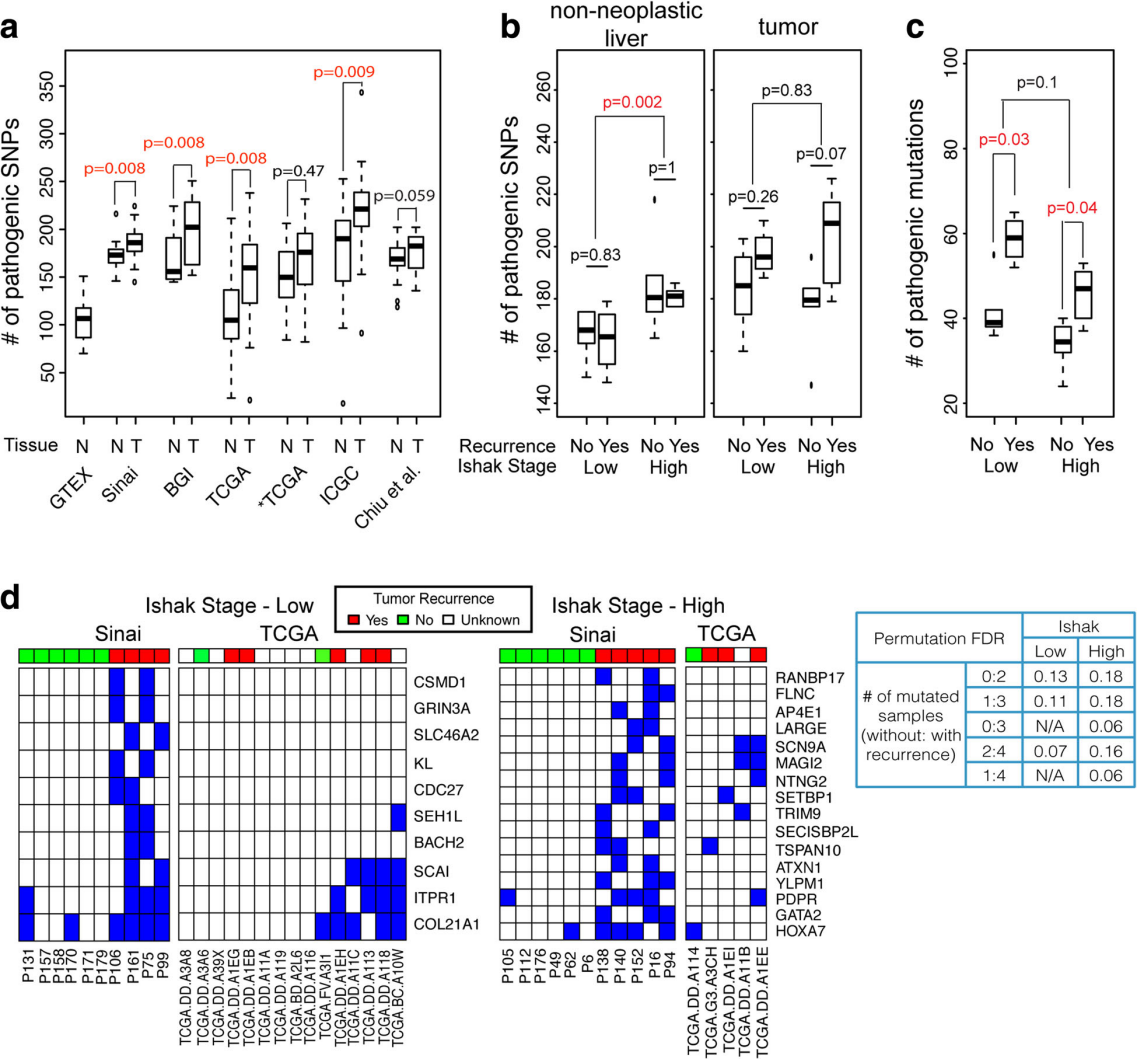
Analysis of SNP variants inferred from GTEx, BGI, and Mount Sinai dataset. **a** The number of potential pathogenic SNPs was compared among GTEx normal liver, non-neoplastic liver, and tumor tissues in Mount Sinai, BGI, TCGA, ICGC, and Chiu et al. [16] datasets. *TCGA indicates the set of seven TCGA samples with HBV integration identified. **b** The number of potential pathogenic SNPs shows a significant association with liver fibrosis in non-neoplastic liver tissues in the Mount Sinai dataset. **c** The number of potential pathogenic somatic mutations is significantly associated with tumor recurrence while it is not significantly associated with liver fibrosis. The difference between the two groups was tested by Wilcoxon rank sum test *P* value. Significant *P* values (*P* < 0.05) are colored in red. **d** Genes with potential pathogenic mutations preferentially occurred in tumor recurrence groups are shown in low and high liver fibrosis groups. Mutated genes are marked in blue. The false discovery rate was assessed by permutation tests. Mutational status of those genes was also analyzed in TCGA samples with and without cirrhosis

Chronic inflammation induced by HBV infection may trigger somatic mutations. Therefore, we investigated whether the number of potential pathogenic mutations in cancer census genes (defined as pathogenic SNP load, Methods) is associated with liver fibrosis stage and tumor recurrence. In order to ensure a fair comparison between normal liver and tumor tissues, we also randomly selected 20 normal liver tissue samples from the GTEx dataset [47] and compared pathogenic SNP loads called for non-neoplastic liver and tumor samples in the Mount Sinai, BGI, TCGA, ICGC, and Chiu et al. [16] datasets. After SNPs were inferred for each sample, we selected those overlapping with pathogenic SNPs curated in the COSMIC dataset [35, 46] (Methods). The pathogenic SNP load was associated with tissue type and increased in the order of normal liver (GTEx), non-neoplastic liver tissues, and tumor (Fig. 6a). The pattern in the Mount Sinai dataset was consistent with results from the BGI, TCGA, ICGC, and Chiu et al. [16] datasets. The pathogenic SNP loads in TCGA non-neoplastic liver tissues were close to the pathogenic SNP loads in normal liver tissues. It is worth noting that HBV integrations were identified in only 7 of 21 pairs of samples in the TCGA HBV-HCC dataset. When considering only pathogenic SNPs in these seven samples (*TCGA in Fig. 6a), the pathogenic SNP load was significantly higher than that in normal liver (Wilcox *P* = 0.005). Genes with pathogenic mutations (Methods) were significantly overlapped with genes with HBV integration in non-neoplastic liver tissues across all datasets (FET *P* = 0.0001, 0.0009, 0.009, and 0.008 for the Mount Sinai, BGI, TCGA, and ICGC dataset, respectively; Additional file 19: Table S11), but not in tumor tissues, suggesting that HBV integrations in non-neoplastic liver tissues and functional somatic mutations target the same set of genes important for tumorigenesis.

When Mount Sinai samples were further separated based on liver fibrosis and tumor recurrence status, there was a significant association between the number of potential pathogenic SNPs and liver fibrosis in non-neoplastic liver tissues (Fig. 6b). Further, pathogenic SNP loads were higher in patients with end-stage fibrosis than in other patients. Pathogenic SNPs and somatic mutations identified in Mount Sinai, TCGA, and ICGC samples with low and high liver fibrosis were significantly overlapped (Additional file 20: Figure S8A, *P* values for overlap are listed in Additional file 21: Table S12). Even though more pathogenic SNPs were identified in tumor tissues, a higher percentage of pathogenic SNPs identified in non-neoplastic liver were common across the three datasets than in tumor tissues in both low fibrosis and cirrhosis groups, suggesting that pathogenic SNPs in non-neoplastic tissues are important in tumorigenesis. Genes with common pathogenic SNPs or mutations were compared with GO biological processes (Additional file 20: Figure S8B). The genes with common pathogenic mutations identified in the non-cirrhosis group were significantly enriched for the biological process response to DNA damage (*P* = 0.0035), but the ones identified in the cirrhosis group were not (*P* = 0.23), suggesting potentially different mechanisms of tumorigenesis in non-cirrhotic and cirrhotic liver.

While the pathogenic SNP load itself was not associated with tumor recurrence status, the number of pathogenic mutations measured by comparing genotype between non-neoplastic liver and tumor tissues was significantly different between tumor recurred and non-recurred patients in both low and high liver fibrosis groups (Fig. 6c), and the number difference of pathogenic mutations between recurred and non-recurred patients was much larger in the low compared to the high fibrosis group, suggesting that different recurrence risk models are needed for patients of low and high fibrosis. We also tested whether the potential pathogenic SNPs and somatic mutations were associated with cccDNA or HBV replicative activity, but no clear differences were observed (Additional file 22: Figure S9, Additional file 3: Supplementary Materials and Methods). Further investigation of pathogenic mutations at gene level identified 10 and 16 genes that were preferentially mutated in the recurrence groups of low and high liver fibrosis, respectively (Fig. 6d, Additional file 23: Table S13). The significance of the bias pattern was assessed by permutations. Several of the genes with mutations that preferentially occurred in the recurrence groups are known for their association with HCC. For example, *COL21A1*, mutated in all four samples in the low fibrosis group, was reported as somatically mutated in two out of a nine intrahepatic metastatic samples in a HBV-HCC cohort [48]. The same study also reported somatic mutations in *CSMD1*, *CDC27*, *SEH1L*, and *ATXN1* in their intrahepatic metastatic samples. *HOXA7*, mostly mutated in the high liver fibrosis group, was reported to promote metastasis of HCC with activation of Snail [49], while decreased expression of GATA2 was correlated with poor prognosis of HCC [50]. In addition, somatic pathogenic mutations related to tumor recurrence in low and high fibrosis identified in the Mount Sinai cohort also occurred in the TCGA dataset (Fig. 6d). For example, three out of five patients of non-cirrhosis with tumor recurrence had pathogenic somatic mutations in *COL21A1*, *ITPR1*, and *SCAI*. However, the information in the TCGA dataset was not sufficient to assess the significance. Considering all of the above, our results suggest that the extent of pathogenic SNPs and/or somatic mutations could provide potential information for HCC recurrence.

## Discussion

HCC recurrence is a significant risk factor for mortality after curative liver resection (Additional file 1: Figure S1). Salvage liver transplantation after HCC recurrence following curative liver resection has inferior overall survival rates compared to primary liver transplantation (no liver resection) for HCC patients [18]. Thus, it is critical to predict which HCC patients have a high risk for recurrence so that they can be treated with adjuvant therapies or considered for liver transplantation prior to surgical resection. Herein, we characterized genomic changes related to HBV infection with regard to HCC recurrence risk. HBV infection induces HBV integration into the host genome and somatic mutations in liver tissue. We examined an HBV insertion and potential pathogenic SNPs in HCC tumor and non-neoplastic liver tissues in HBV-HCC patients of low or high liver fibrosis stage. Recently, Zhao et al. [11] reported distinct patterns of HBV integration host genes in cirrhosis-dependent HCC, but our study is the first to suggest that potentially different tumorigenesis mechanisms underlie tumor recurrence in patients with varying liver fibrosis stages.

To accurately identify HBV integration events of low IAF, we developed a pipeline based on VirusFinder. Our results showed that there were more HBV integration sites in the non-neoplastic liver tissues than in tumor tissues, suggesting that selective clone expansion occurs during tumorigenesis (Fig. 4a). This observation is consistent with results from the validation datasets (BGI, TCGA, and ICGC datasets) analyzed through our pipeline and from previously reported studies [15, 16] (Additional file 12: Figure S5). However, they contradict those from other studies reporting more integration sites in tumor tissues [11, 13, 14], likely due to the low sequence coverage in DNA sequencing in the datasets. It is worth noting that our results were based on RNAseq data, which had higher depth of coverage than the common depth of the available WGS data.

HBV integration does not occur at random sites, but tends to occur in regions with active transcription and with repetitive sequences [15, 51]. For example, herein, HBV integration in *FN1* occurred preferentially in patients with high liver fibrosis stage to in those with low fibrosis stage. Fibronectin is an abundantly expressed transcript in non-neoplastic liver, and its expression increases during liver fibrogenesis [52]. Around the virus integration sites, human and virus genome sequences are likely similar, termed as microhomologous (MH) [53], and MH-mediated DNA repair may be a main mechanism mediating virus integration processes [53]. MH sequences between the human and virus genomes are significantly enriched near integration breakpoints for HPV [53] and HBV [11, 13, 53]. We collected flanking regions at the HBV integration sites and compared background numbers of a specific MH size (Additional file 3: Supplementary Materials). MH sequences were enriched near HBV integration sites in our dataset, and the enrichment was significant for a MH size of 2 and 5 bp (Additional file 24: Figure S10A). To the best of our knowledge, this is the first RNAseq-based study replicating the MH enrichment observed in previous studies [11]. We also compared HBV integration sites with regards to CpG islands and common/rare fragile sites [54] (Additional file 3: Supplementary Materials and Methods), and observed no enrichment over that expected by chance (Additional file 24: Figure S10B and S10C).

We identified a few host genes recurrently targeted by HBV integration, which overlapped with several HBV host genes previously reported, including *ALB*, *KMT2B*, *FN1*, and *TERT*. Our study also identified many novel HBV fusion transcripts such as *ARAP2*, *PRKCE*, and *TCF4*. HBV integration in *ARAP2* occurred in two patients, both with integration within the promoter region, and was associated with lower expression in non-neoplastic liver (Additional file 17: Figure S7B). Interestingly, the two patients with HBV integration in *ARAP2* both had end-stage liver fibrosis and cancer recurrence. *ARAP2* is known to regulate focal adhesion dynamics that connect the actin cytoskeleton with the extracellular matrix [55]. While its functions suggest potential roles in tumor progression and metastasis, no previous implications between *ARAP2* and HBV-HCC have been reported. Changes in *TCF4* expression have been linked to tumor progression through stimulation of the Wnt pathway [56]. However, it has not been shown that *TCF4* can be a direct target of the virus. *PRKCE* was one of the most frequently targeted fusion transcripts in non-neoplastic liver (5/21, 24%). It is worth noting that HBV insertion locations in these five samples were identical at chr2:46344574, which is located at intron 11 of the gene. *PRKCE*, protein kinase C, is a tumor suppressor gene involved in apoptosis [57].

In non-neoplastic tissues of both low and high liver fibrosis host genes with HBV insertion in patients with cancer recurrence were enriched for tumor suppressor genes (Fig. 5a), suggesting that non-neoplastic tissues contain information for potential cancer recurrence. These results indicate that both the number of integration events and specific host genes with HBV insertion are critical for tumor recurrence.

Our results also suggested that the number of potential pathogenic SNP gains in tumor over non-neoplastic liver tissues were significantly associated with tumor recurrence in patients of both low and high liver fibrosis (Fig. 6c). Genotypes from non-neoplastic tissues of HBV-HCC patients may be different from germline genotypes (Fig. 6a). To investigate whether the number of pathogenic SNP gains over germline genotype in either tumor or non-neoplastic tissues is associated with tumorigenesis and tumor recurrence, germline genotypes measured in tissues not affected by HBV are needed. It is interesting that genes with pathogenic somatic mutations significantly overlap with HBV fusion host genes in non-neoplastic liver tissues (Additional file 19: Table S11), indicating that both HBV integration and mutations might target a similar set of genes for tumorigenesis. All our analyses results consistently suggest that transcripts with HBV integration and pathogenic SNPs in non-neoplastic liver tissues carry important information of tumorigenesis potential. Accumulation of a few pathogenic somatic mutations on top of these pathogenic SNPs and HBV fusion transcripts may lead to tumorigenesis. However, the potential to accumulate critical somatic mutations may reflect in genomic features in non-neoplastic liver tissues.

Of note, the sample size in the current study was small. Further studies of large sample sizes are needed to validate the associations between HBV-HCC recurrence and HBV integration patterns and/or pathogenic SNP loads. However, compared with the two largest TCGA and ICGA liver cancer sequencing studies, the sample size of our HBV-HCC RNA sequencing study was similar (Additional file 2: Table S1) and the clinical follow-up was more comprehensively recorded herein. Importantly, our study was designed with balanced groups in terms of fibrosis stage and tumor recurrence such that the potential tumorigenesis mechanism differences between high and low liver fibrosis groups could be assessed. Regardless of the sample size limitation, our findings are consistent with results from other independent dataset such as BGI, TCGA, ICGC, and Chiu et al. [16] HBV-HCC cohorts. The association between HBV insertion events and intrahepatic HBV replicative activity suggests that a potential approach to prevent HBV-HCC recurrence is to continuously administer anti-HBV drugs following tumor resection; however, further testing of this in formal clinical trials is needed.

## Conclusions

We performed systematic comparison of molecular features of HBV-HCC patients with low- and high-degree of liver fibrosis. The results suggest that HBV integrations and pathogenic SNPs in non-neoplastic tissues are important for tumorigenesis and different recurrence risk models are needed for patients with low and high degrees of liver fibrosis. Further study of larger sample size will shed more light on molecular mechanisms underlying differences between two groups of patients.

## Additional files

Additional file 1: Figure S1. Overall survival associated with tumor recurrence after HCC resection. (TIF 403 kb)

Additional file 2: Table S1. Clinical information of HBV-HCC samples in Mount Sinai, TCGA, and ICGC cohorts. (XLSX 403 kb)

Additional file 3: Supplementary materials and methods. (ZIP 1597 kb)

Additional file 4: Table S2. Comparison of cirrhotic signatures with other liver cancer signatures in MSigDB. (XLSX 2020 kb)

Additional file 5: Figure S2. Differentially expressed genes signatures. (A) Differentially expressed genes between low and high liver fibrosis group are shown in heatmap. (B) Heatmap of 186 prognostic signatures genes from Hoshida et al. [38]. (TIF 1610 kb)

Additional file 6: Figure S3. Detail mapping of human and HBV genome on missing HBV integration sites reported in Sung et al. [13]. For each BGI HBV integration site not identified by our method, partial aligned sequences were colored in red and blue for human and virus, respectively. (TIF 950 kb)

Additional file 7: Table S3. Summary of number of HBV integration sites in BGI samples. (XLSX 45 kb)

Additional file 8: Table S4. Comparison of HBV integration sites reported by Sinai and TCGA. (XLSX 48 kb)

Additional file 9: Table S5. HBV integration sites identified in Mount Sinai cohort. (XLSX 64 kb)

Additional file 10: Figure S4. Association between serum HBsAg level and the number of human transcripts with HBV integration. (A) Serum HBsAg level (IU/ml_log) and the number of human transcripts with HBV S ORF integrated were significantly associated in both non-neoplastic liver and tumor tissue. (B) Serum HBsAg level was marginally associated with the number of all human transcripts with HBV integration in non-neoplastic liver tissues, but significantly associated with the number of all HBV integrated human transcripts in tumor tissues. The association was measured by Spearman correlation coefficient (rho) and the *P* value of the rho. (TIF 450 kb)

Additional file 11: Table S6. Transcripts with recurrent HBV integration. (XLSX 50 kb)

Additional file 12: Figure S5. Comparison of the number of HBV fusion transcripts in non-tumor and tumor tissue in multiple HBV-HCC dataset. HBV fusion genes were identified based on our method for Mount Sinai, BGI, TCGA, and ICGC datasets, and those by Chiu et al. [16] and Jhunjhunwala et al. [12] were reported in their own studies. (TIF 195 kb)

Additional file 13: Figure S6. Characterization of HBV integration events. (A) Distribution of HBV breakpoints in HBV integration. The number of HBV integration events was counted within each bin of 100 bases. The common known breakpoint, nt1818 is marked with a red dashed line. (B) Transcriptome coverage of RNAseq dataset. For the dataset used in our study, we measured the ratio of intron/exon in our RNAseq data. (C) Distribution of genomic preferences of HBV integration in other datasets. HBV integration sites were identified using our pipeline (BGI and TCGA). (TIF 491 kb)

Additional file 14: Table S7. HBV integration sites identified in BGI, TCGA, and ICGC cohorts. (XLSX 92 kb)

Additional file 15: Table S8. GO analysis with HBV fusion transcripts in Mount Sinai cohort. (XLSX 65 kb)

Additional file 16: Table S9. Comparison of HBV fusion transcripts with cancer-related genes. (XLSX 37 kb)

Additional file 17: Figure S7. Gene expression influenced by HBV integration. For the recurrent host genes, the gene expression is compared between samples with and without integration. Two recurrent host genes, (A) *KMT2B* and (B) *ARAP2*, show gene expression changes induced by HBV integrations. *P* value is measured by the Student *t*-test. (C) Differentially expressed genes between tumors with and without HBV-KMT2B integration. A total of 139 genes were over-expressed in the tumors with HBV-KMT2B integration while 32 were under-expressed. The list of the top 20 in over-expressed (red) and top 5 under expressed (green) enriched GO terms within each gene set is shown. (TIF 1420 kb)

Additional file 18: Table S10. HBV fusion transcript enrichment for tumor suppressor genes in different liver fibrosis and tumor recurrent status. (XLSX 38 kb)

Additional file 19: Table S11. Association between pathogenic mutations and HBV integration host genes. (XLSX 41 kb)

Additional file 20: Figure S8. Overlaps among pathogenic SNPs and mutations identified in Mount Sinai, TCGA, and ICGC datasets. (A) Common pathogenic SNPs and mutations in non-neoplastic liver and tumor tissues with and without cirrhosis. (B) The heatmap of GO analysis based on the common pathogenic SNPs or mutations among three datasets. (TIF 577 kb)

Additional file 21: Table S12. Common pathogenic SNPs and somatic mutations among different datasets. (XLSX 47 kb)

Additional file 22: Figure S9. Association of pathogenic variants with cccDNA. (A) cccDNA and (B) HBV replicative activity with pathogenic SNPs. (C) cccDNA and (D) HBV replicative activity with pathogenic mutations. (TIF 556 kb)

Additional file 23: Table S13. Pathogenic somatic mutations biased in tumor recurrence group. (XLSX 10 kb)

Additional file 24: Figure S10. HBV integration preference in specific genomic regions. (A) Microhomologs between human and HBV (B) CpG sites (islands, shore, and shelf), and (C) genomic fragile sites (common and rare). χ^2^ *P* value measures relationship between HBV integration and specific features. (TIF 660 kb)

## Abbreviations

FET: Fisher’s exact test
HBV: hepatitis B virus
HCC: hepatocellular carcinoma
IAF: insertion allele frequency
WGS: whole genome sequencing
