# Supplementary figures and images for: A pilot systematic genomic comparison of recurrence risks of hepatitis B virus-associated hepatocellular carcinoma with low- and high-degree liver fibrosis
Source: BMC Med. 2017 Dec 7;15:214. doi: 10.1186/s12916-017-0973-7 (PMC5719570; doi:10.1186/s12916-017-0973-7)

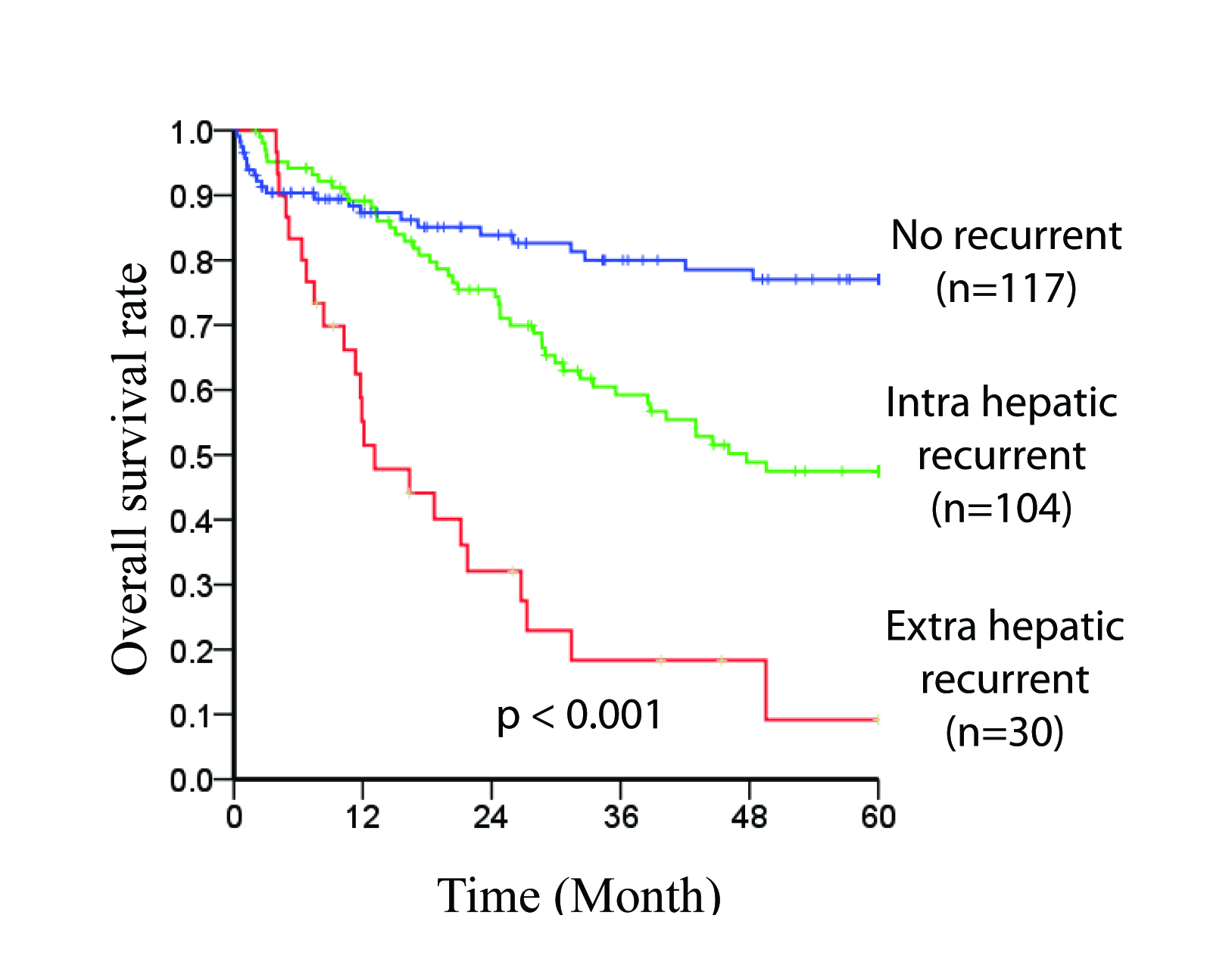

Supplement: Supplementary file 1 — Overall survival associated with tumor recurrence after HCC resection. (TIF 403 kb) [file 12916_2017_973_MOESM1_ESM.tif]

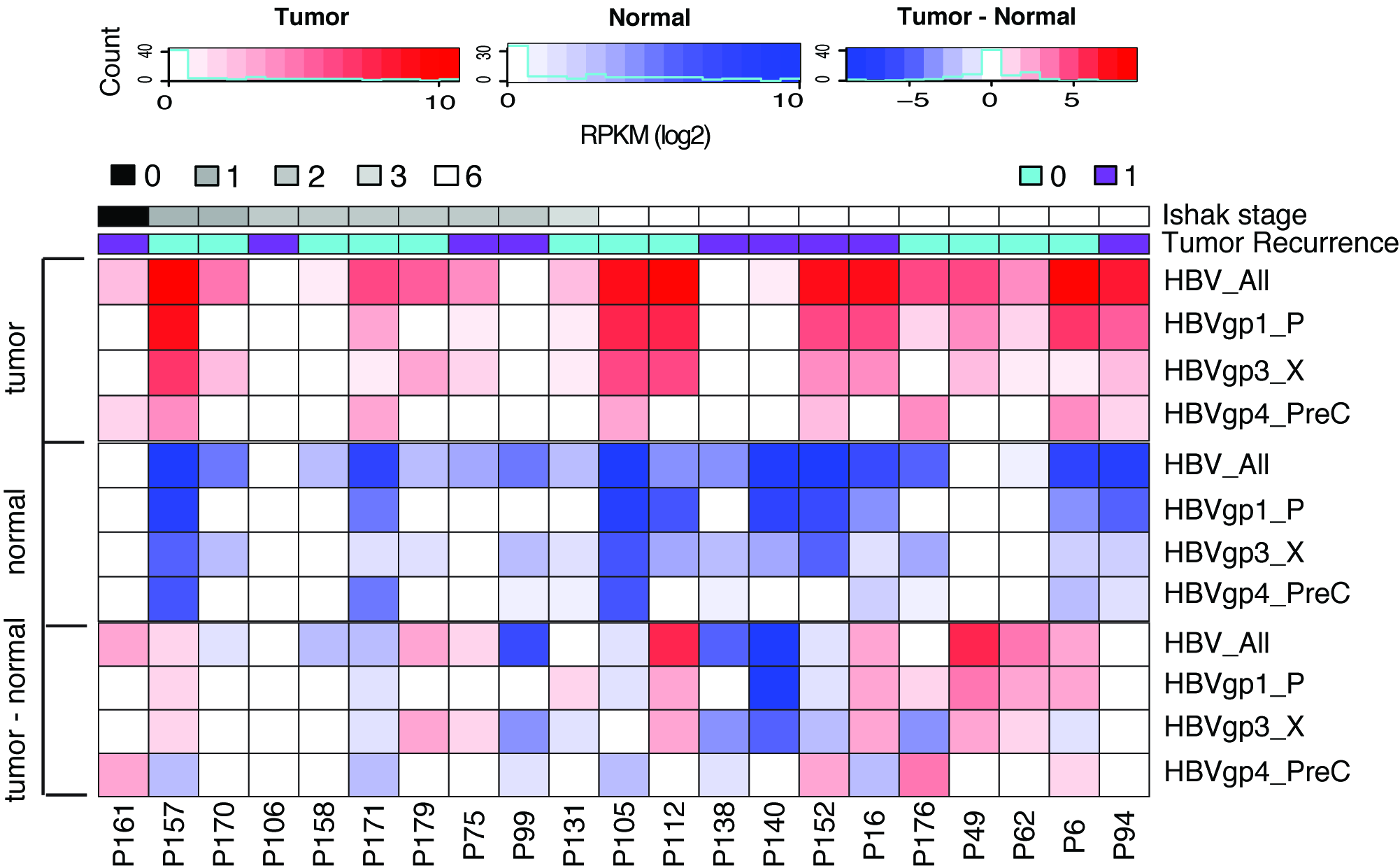

Supplement: Supplementary file 3 — Supplementary materials and methods. (ZIP 1597 kb) [file 12916_2017_973_MOESM3_ESM.zip › Figure S11.tif]

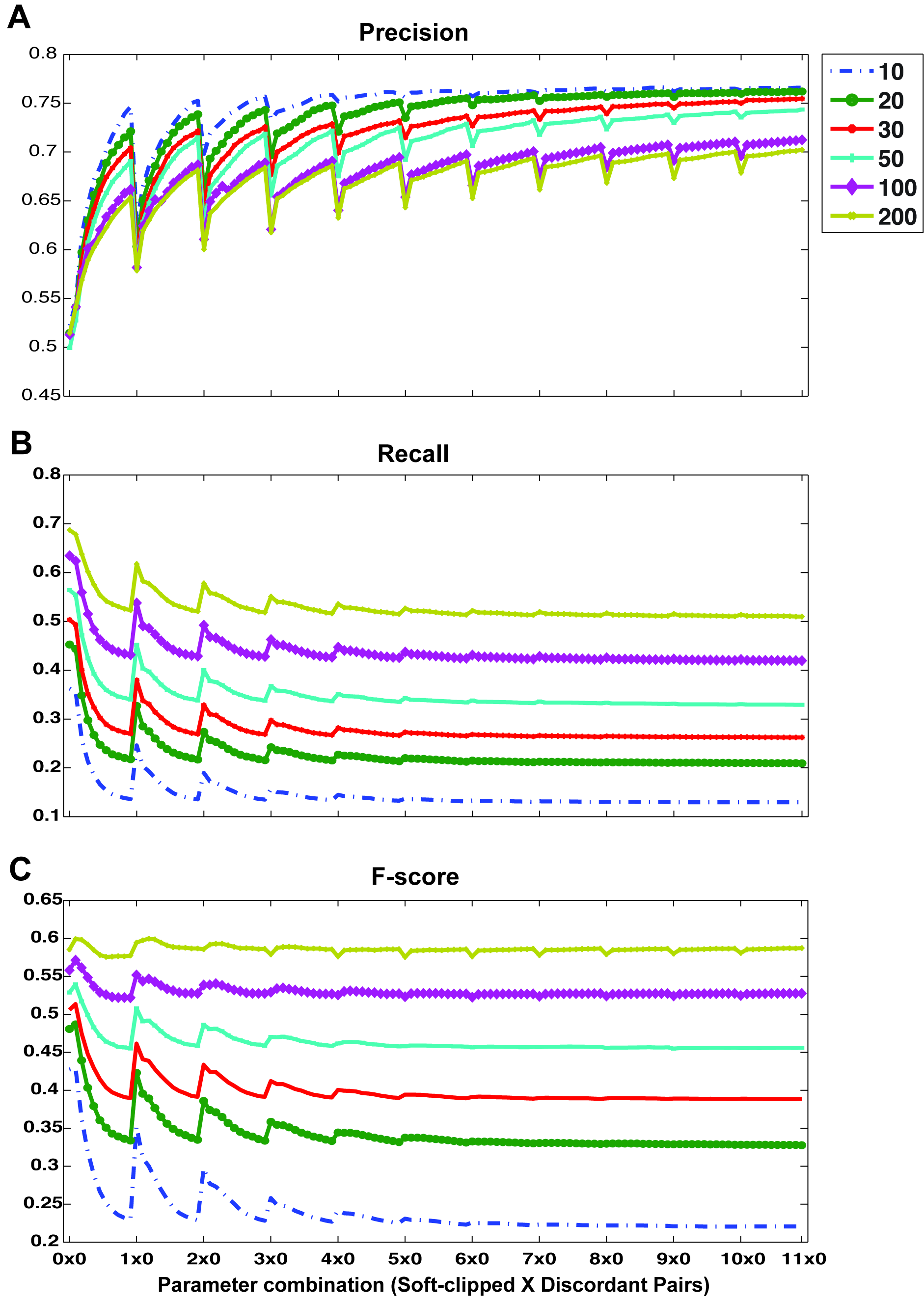

Supplement: Supplementary file 3 — Supplementary materials and methods. (ZIP 1597 kb) [file 12916_2017_973_MOESM3_ESM.zip › Figure S12.tif]

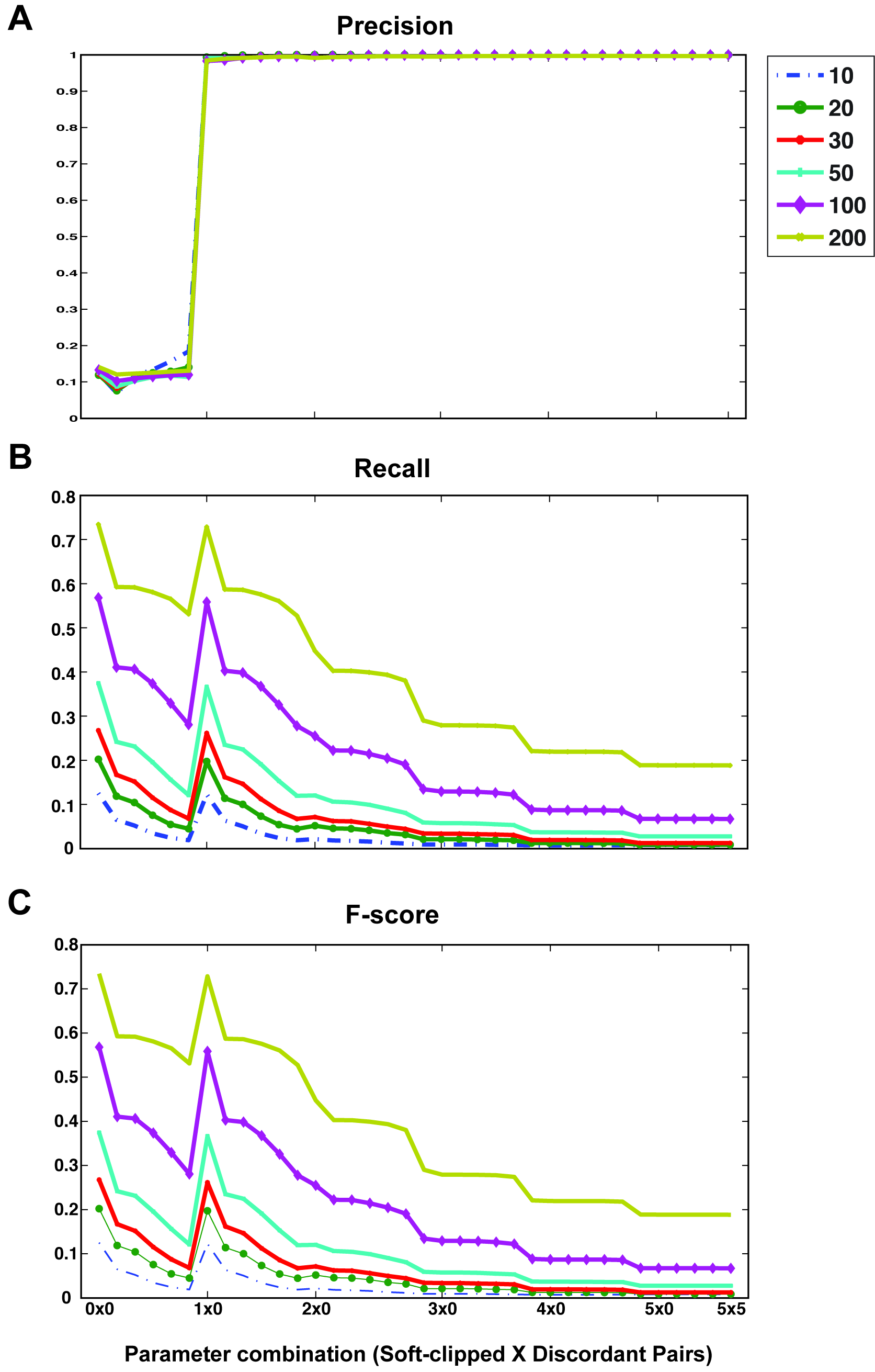

Supplement: Supplementary file 3 — Supplementary materials and methods. (ZIP 1597 kb) [file 12916_2017_973_MOESM3_ESM.zip › Figure S13.tif]

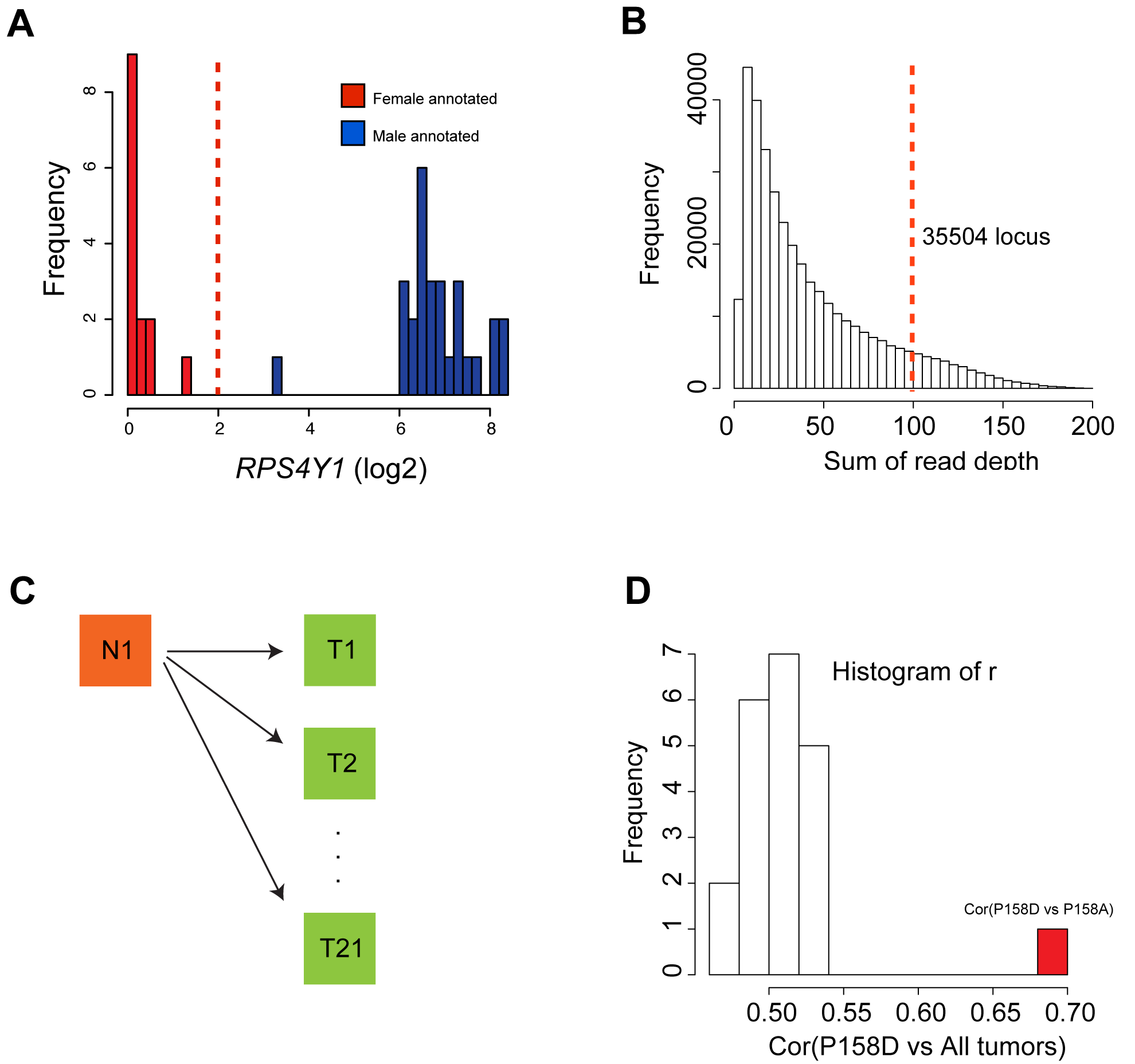

Supplement: Supplementary file 3 — Supplementary materials and methods. (ZIP 1597 kb) [file 12916_2017_973_MOESM3_ESM.zip › Figure S14.tif]

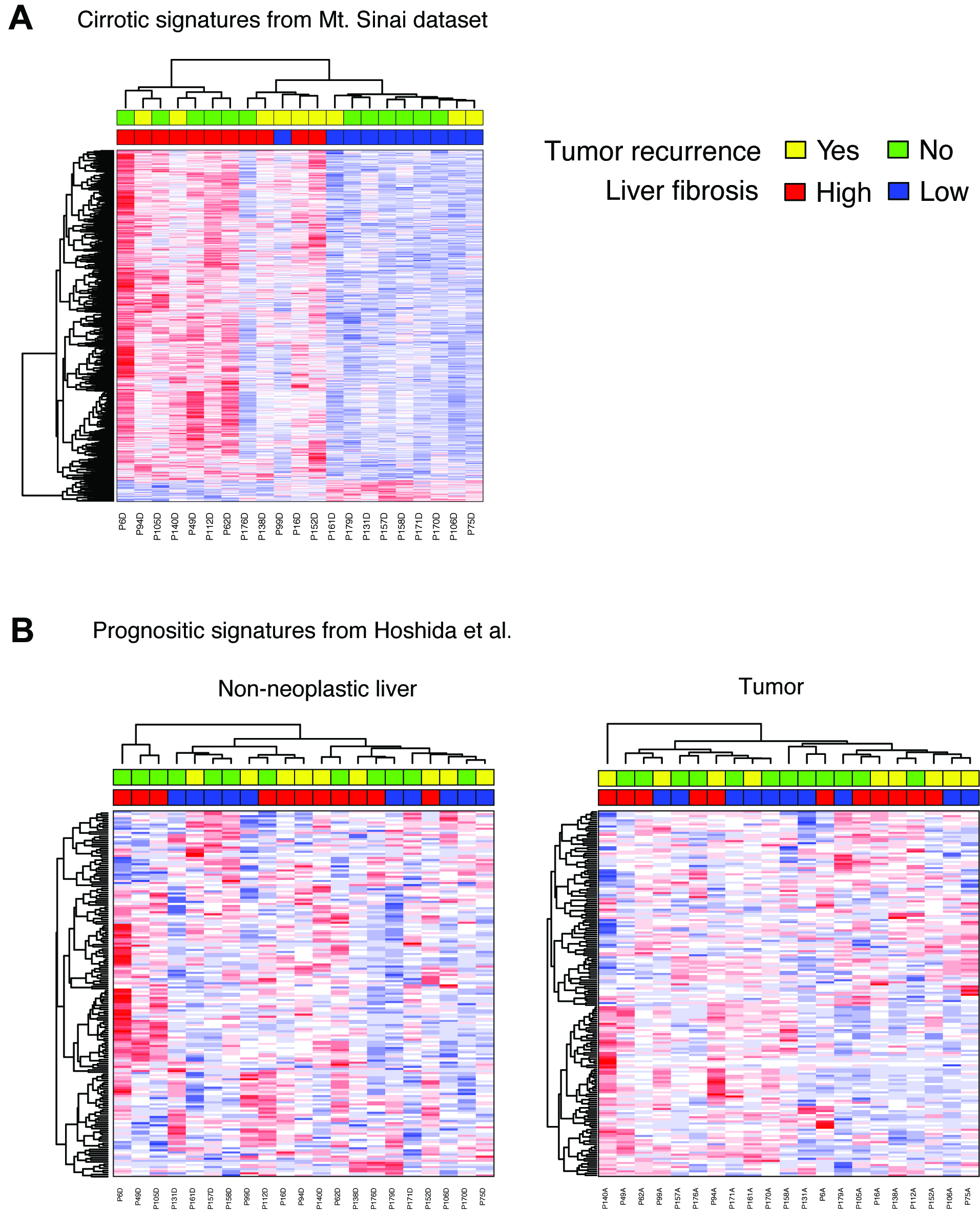

Supplement: Supplementary file 5 — Differentially expressed genes signatures. (A) Differentially expressed genes between low and high liver fibrosis group are shown in heatmap. (B) Heatmap of 186 prognostic signatures genes from Hoshida et al. [38]. (TIF 1610 kb) [file 12916_2017_973_MOESM5_ESM.tif]

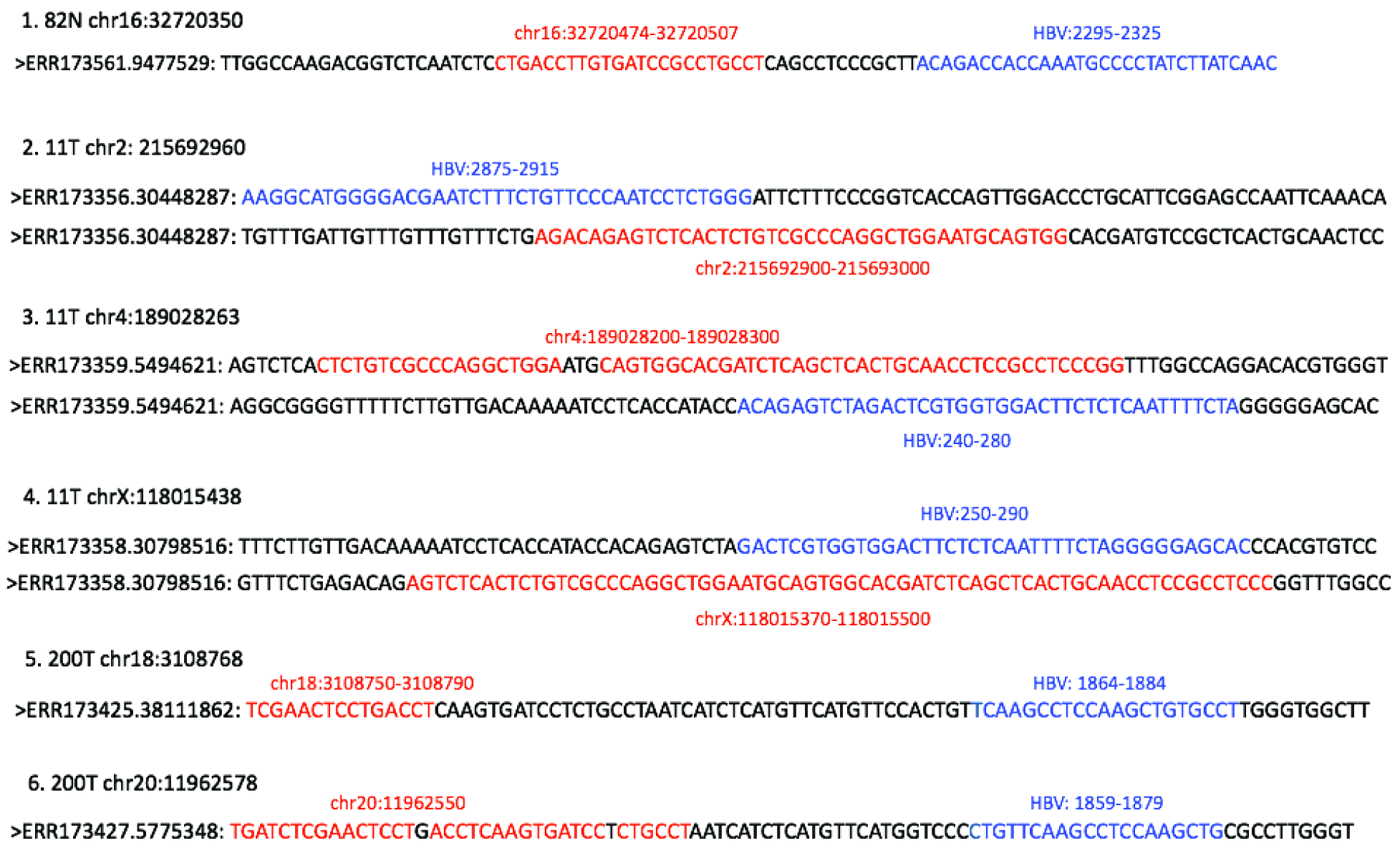

Supplement: Supplementary file 6 — Detail mapping of human and HBV genome on missing HBV integration sites reported in Sung et al. [13]. For each BGI HBV integration site not identified by our method, partial aligned sequences were colored in red and blue for human and virus, respectively. (TIF 950 kb) [file 12916_2017_973_MOESM6_ESM.tif]

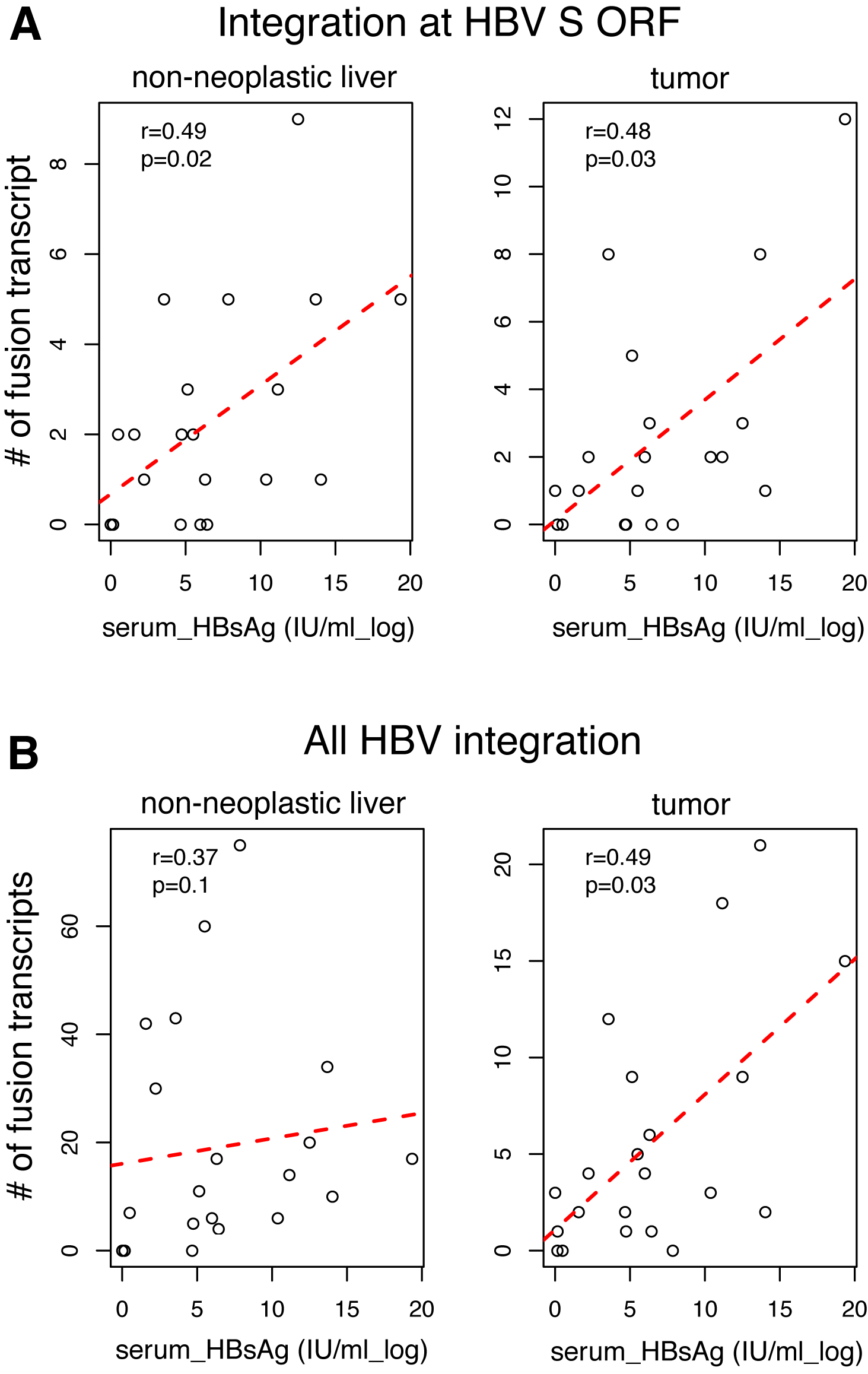

Supplement: Supplementary file 10 — Association between serum HBsAg level and the number of human transcripts with HBV integration. (A) Serum HBsAg level (IU/ml_log) and the number of human transcripts with HBV S ORF integrated were significantly associated in both non-neoplastic liver and tumor tissue. (B) Serum HBsAg level was marginally associated with the number of all human transcripts with HBV integration in non-neoplastic liver tissues, but significantly associated with the number of all HBV integrated human transcripts in tumor tissues. The association was measured by Spearman correlation coefficient (rho) and the P value of the rho. (TIF 450 kb) [file 12916_2017_973_MOESM10_ESM.tif]

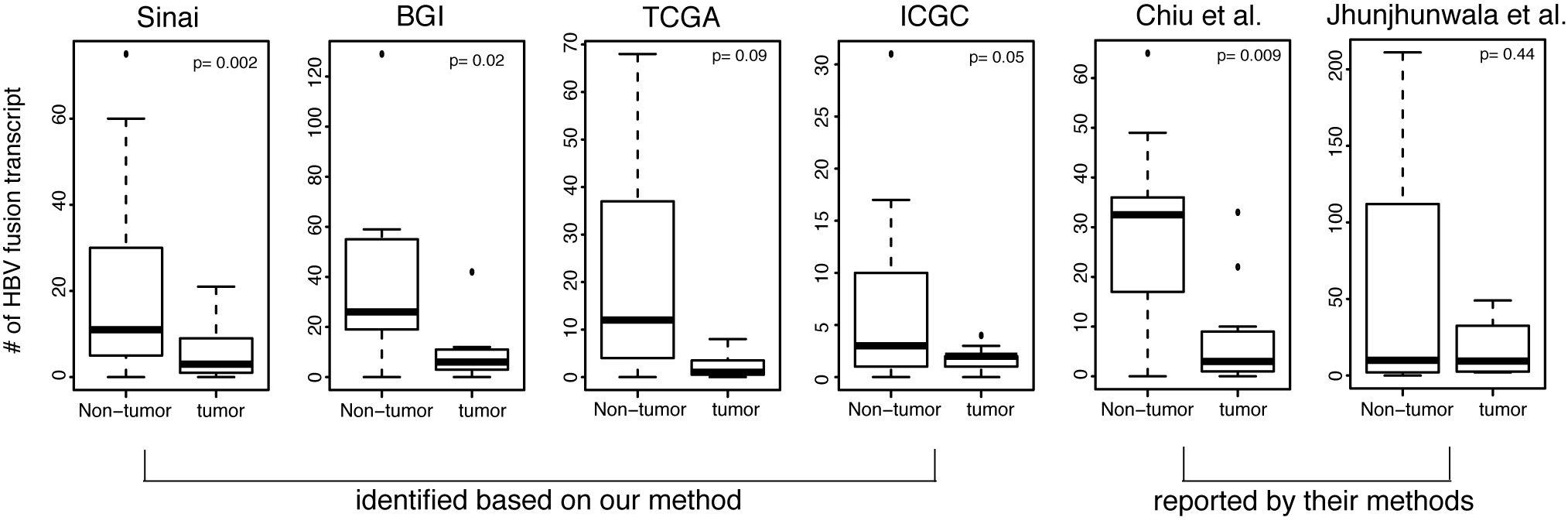

Supplement: Supplementary file 12 — Comparison of the number of HBV fusion transcripts in non-tumor and tumor tissue in multiple HBV-HCC dataset. HBV fusion genes were identified based on our method for Mount Sinai, BGI, TCGA, and ICGC datasets, and those by Chiu et al. [16] and Jhunjhunwala et al. [12] were reported in their own studies. (TIF 195 kb) [file 12916_2017_973_MOESM12_ESM.tif]

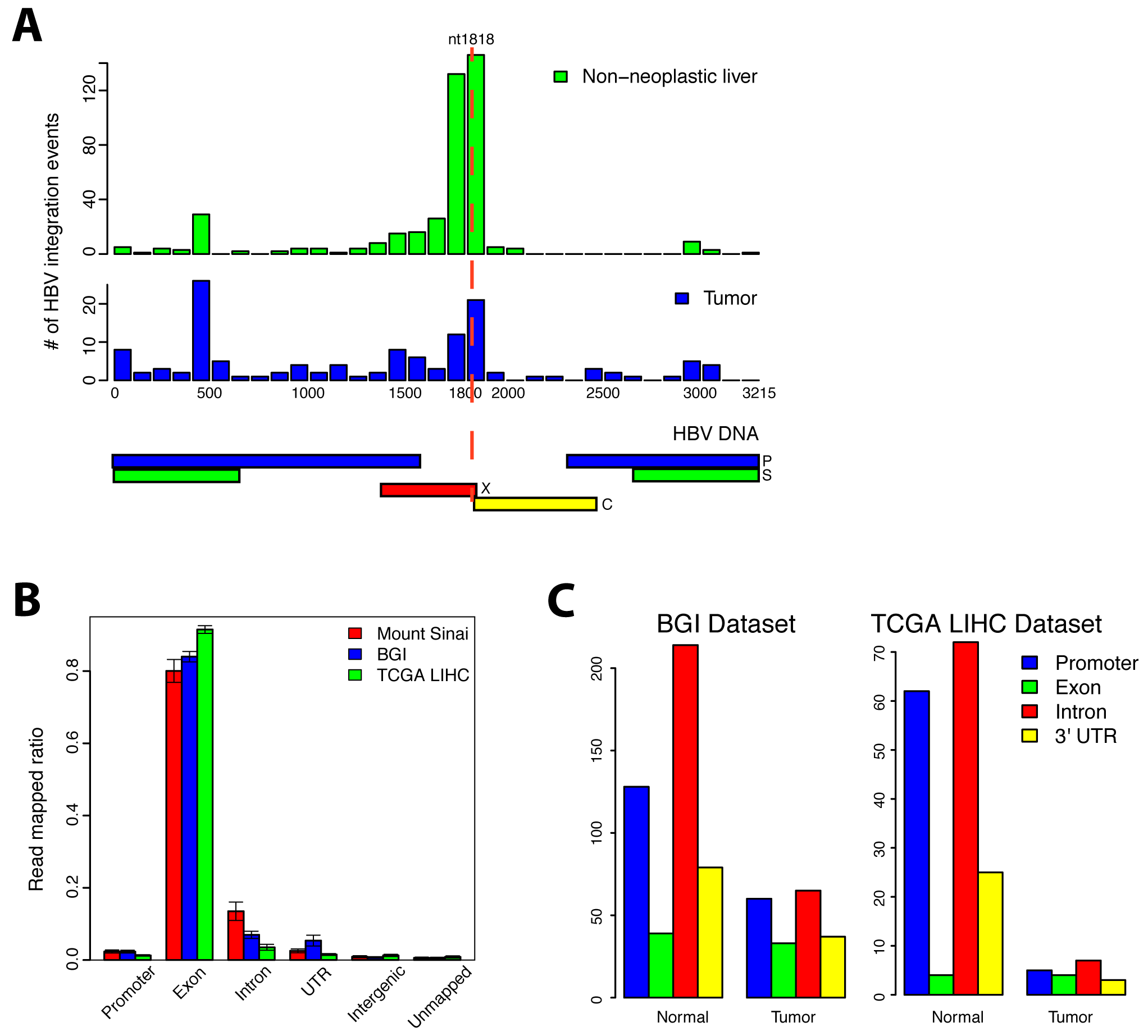

Supplement: Supplementary file 13 — Characterization of HBV integration events. (A) Distribution of HBV breakpoints in HBV integration. The number of HBV integration events was counted within each bin of 100 bases. The common known breakpoint, nt1818 is marked with a red dashed line. (B) Transcriptome coverage of RNAseq dataset. For the dataset used in our study, we measured the ratio of intron/exon in our RNAseq data. (C) Distribution of genomic preferences of HBV integration in other datasets. HBV integration sites were identified using our pipeline (BGI and TCGA). (TIF 491 kb) [file 12916_2017_973_MOESM13_ESM.tif]

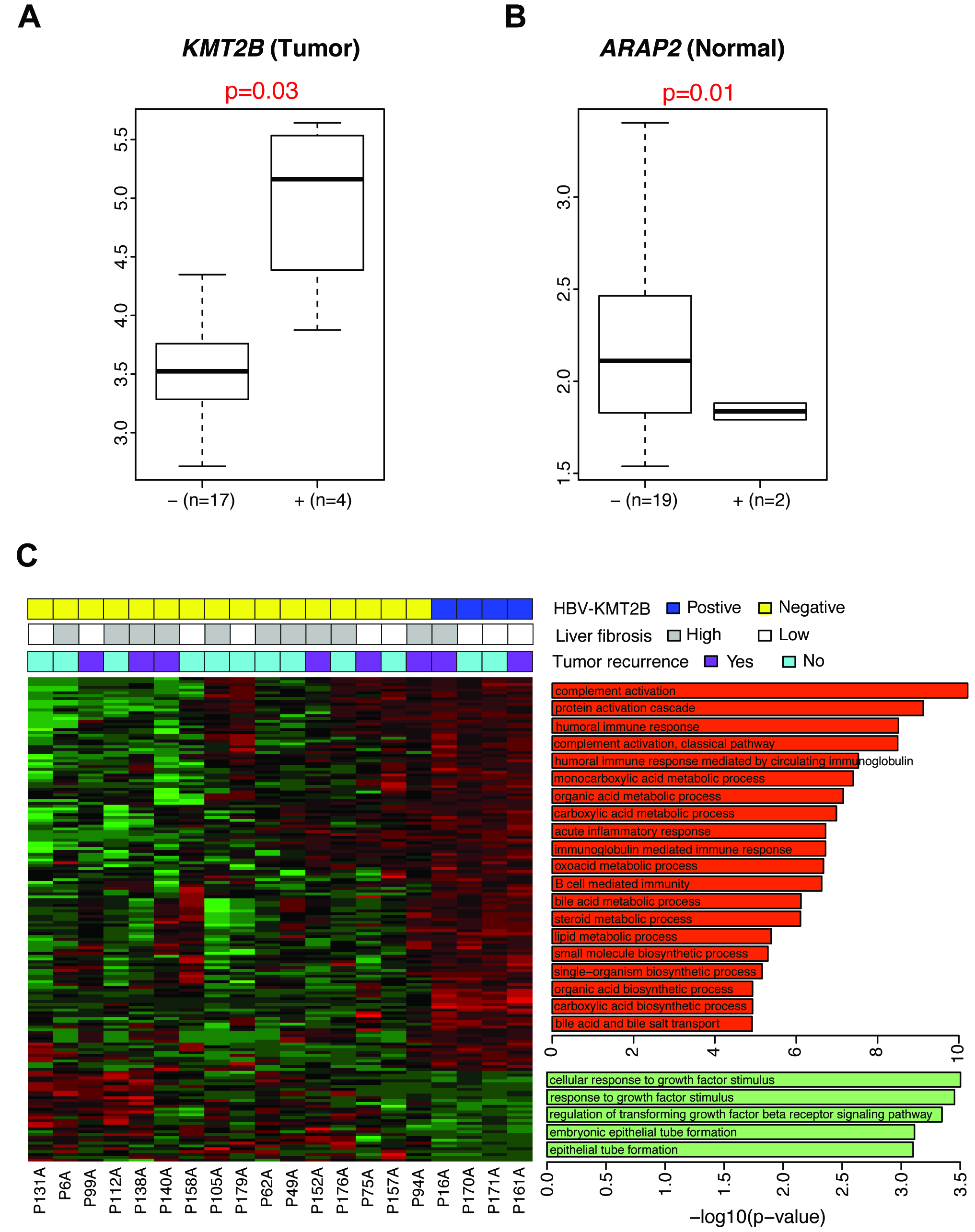

Supplement: Supplementary file 17 — Gene expression influenced by HBV integration. For the recurrent host genes, the gene expression is compared between samples with and without integration. Two recurrent host genes, (A) KMT2B and (B) ARAP2, show gene expression changes induced by HBV integrations. P value is measured by the Student t-test. (C) Differentially expressed genes between tumors with and without HBV-KMT2B integration. A total of 139 genes were over-expressed in the tumors with HBV-KMT2B integration while 32 were under-expressed. The list of the top 20 in over-expressed (red) and top 5 under expressed (green) enriched GO terms within each gene set is shown. (TIF 1420 kb) [file 12916_2017_973_MOESM17_ESM.tif]

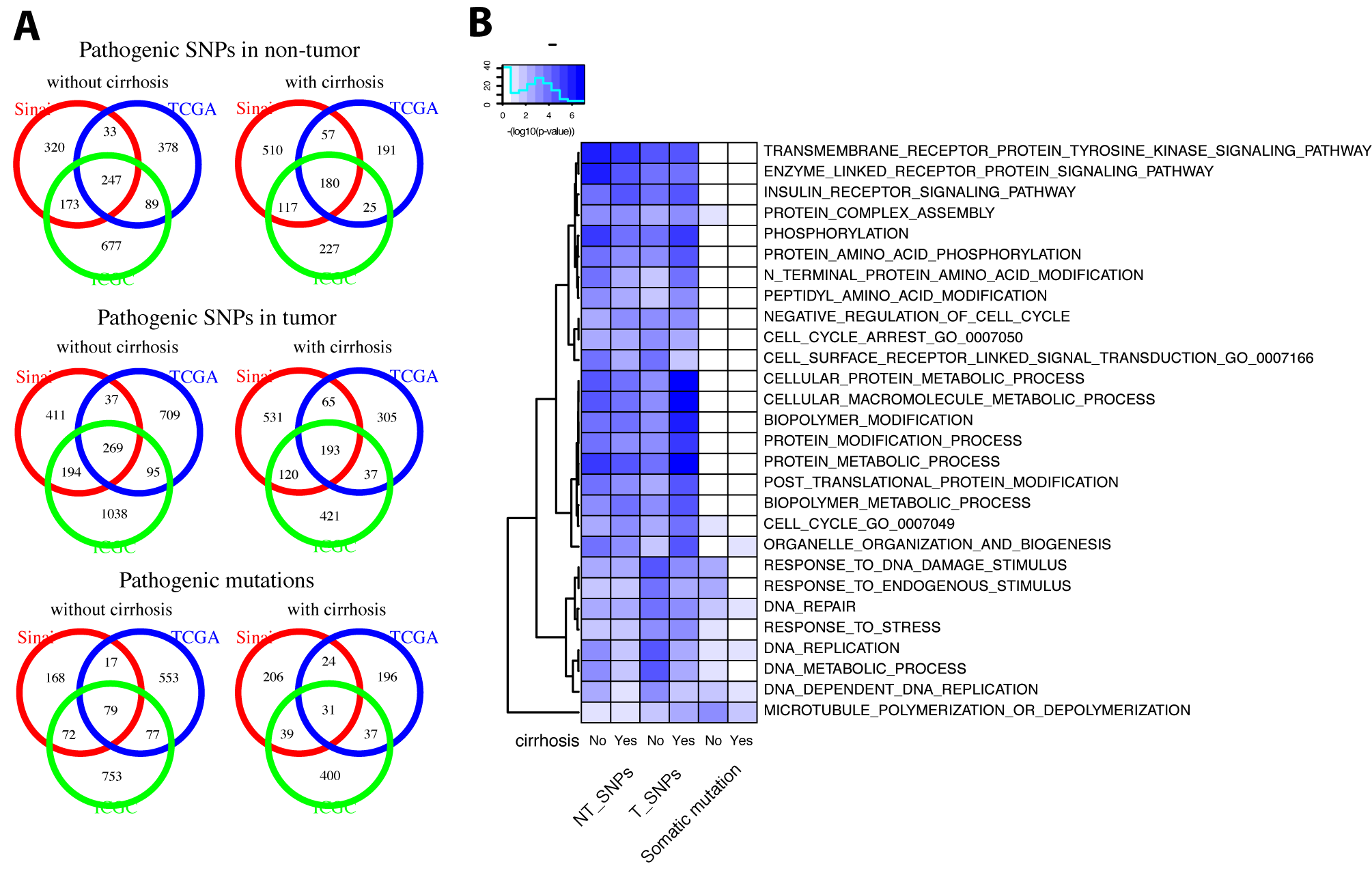

Supplement: Supplementary file 20 — Overlaps among pathogenic SNPs and mutations identified in Mount Sinai, TCGA, and ICGC datasets. (A) Common pathogenic SNPs and mutations in non-neoplastic liver and tumor tissues with and without cirrhosis. (B) The heatmap of GO analysis based on the common pathogenic SNPs or mutations among three datasets. (TIF 577 kb) [file 12916_2017_973_MOESM20_ESM.tif]

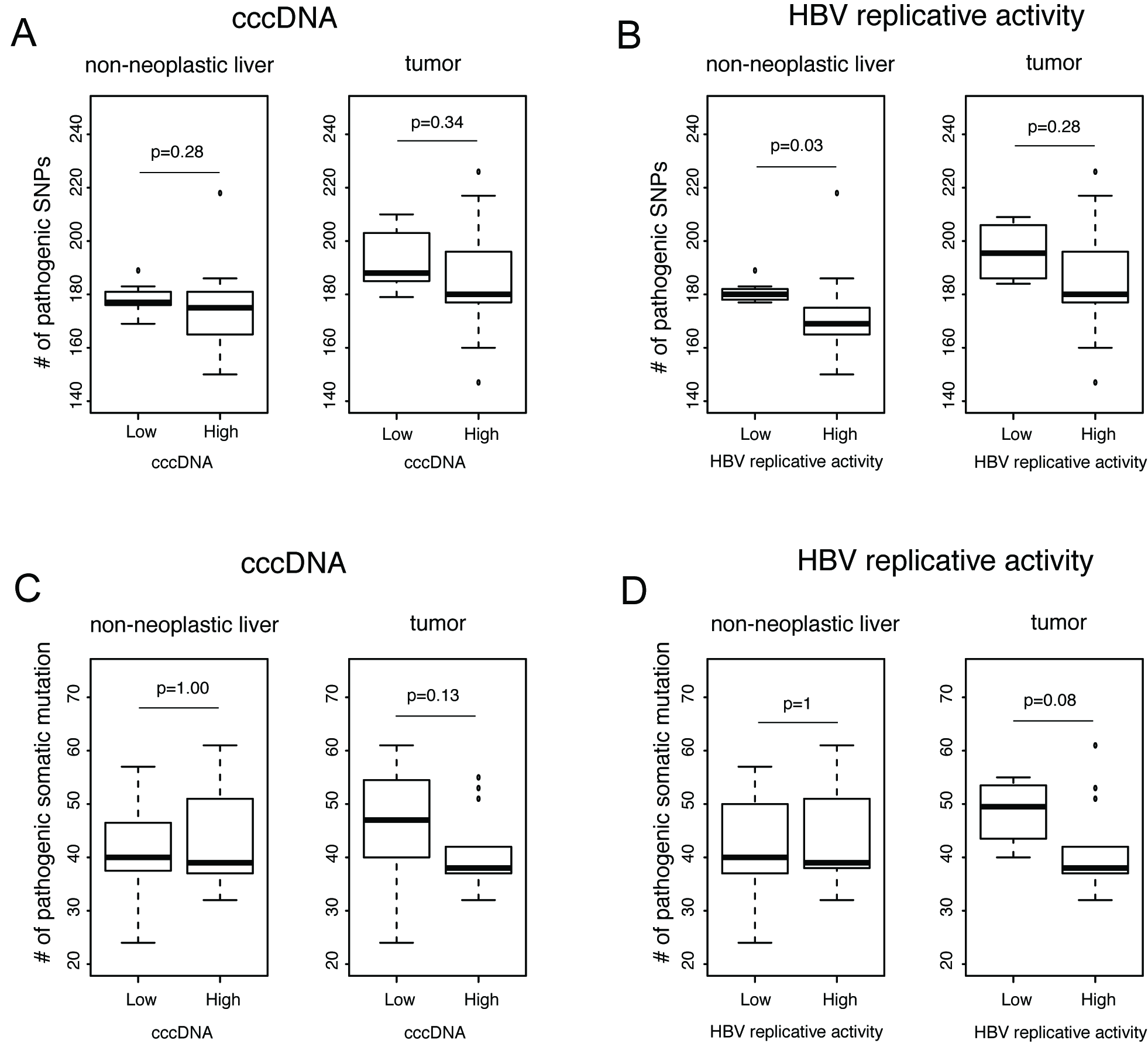

Supplement: Supplementary file 22 — Association of pathogenic variants with cccDNA. (A) cccDNA and (B) HBV replicative activity with pathogenic SNPs. (C) cccDNA and (D) HBV replicative activity with pathogenic mutations. (TIF 556 kb) [file 12916_2017_973_MOESM22_ESM.tif]

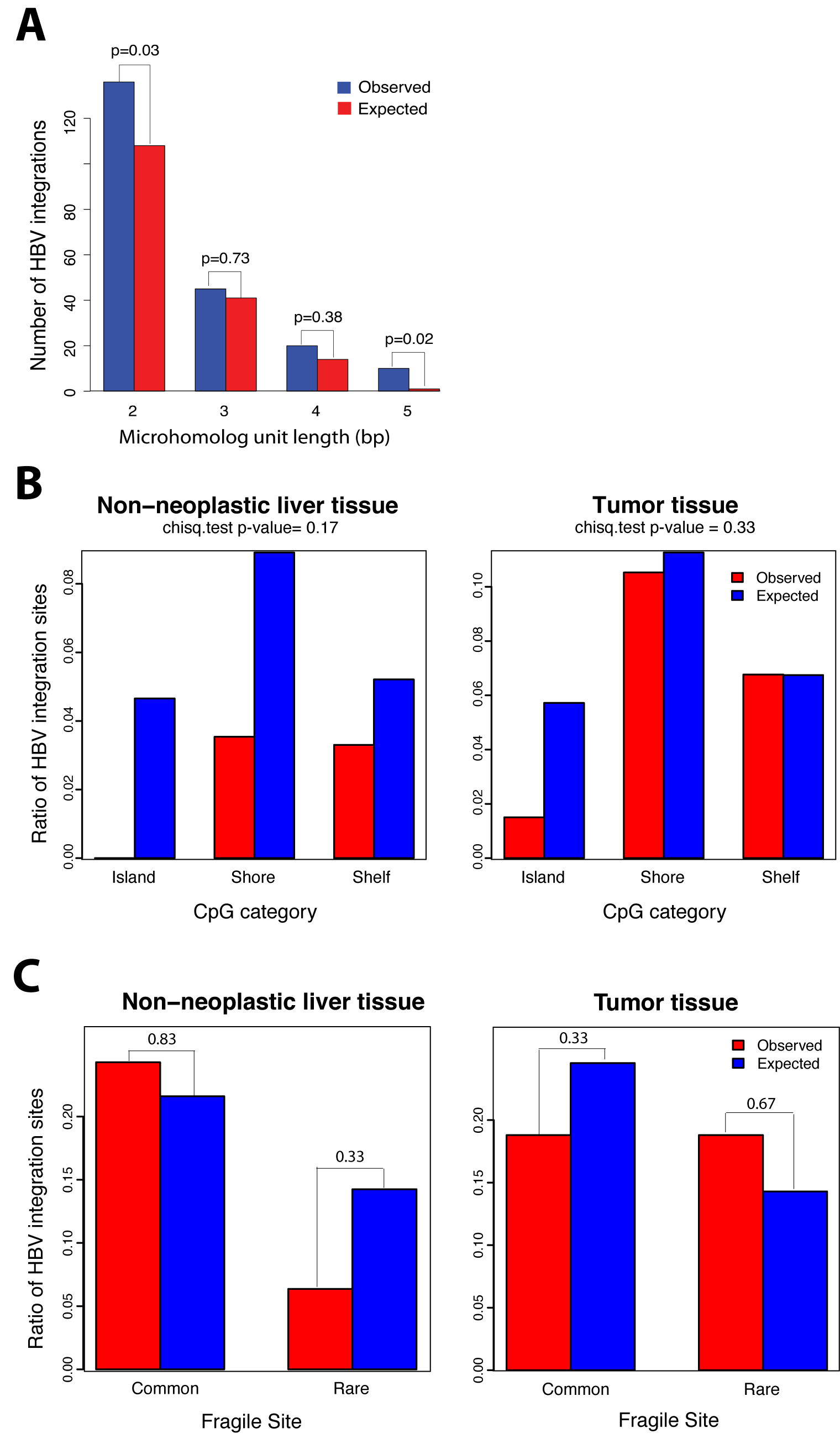

Supplement: Supplementary file 24 — HBV integration preference in specific genomic regions. (A) Microhomologs between human and HBV (B) CpG sites (islands, shore, and shelf), and (C) genomic fragile sites (common and rare). χ2 P value measures relationship between HBV integration and specific features. (TIF 660 kb) [file 12916_2017_973_MOESM24_ESM.tif]
