# Supplementary material for: A pilot systematic genomic comparison of recurrence risks of hepatitis B virus-associated hepatocellular carcinoma with low- and high-degree liver fibrosis
Source: BMC Med. 2017 Dec 7;15:214. doi: 10.1186/s12916-017-0973-7 (PMC5719570; doi:10.1186/s12916-017-0973-7)
Supplement: Supplementary file 3 — Supplementary materials and methods. (ZIP 1597 kb) [file 12916_2017_973_MOESM3_ESM.zip › Supplementary_materials_methods_R2_final.docx]

**Supplementary Materials and Methods for**

**A Pilot Systematic Genomic Comparison of Recurrence Risks of Hepatitis B Virus-associated Hepatocellular Carcinoma with low and high degree of liver fibrosis**

Author names:

Seungyeul Yoo^1,2†^, Wenhui Wang^1,2†^, Qin Wang^3†^, Maria I Fiel^4^, Eunjee Lee^1,2,5^, Spiros P. Hiotis^3*^, Jun Zhu^1,2,5*^

Author institutions:

1. Department of Genetics and Genomic Sciences, Icahn School of Medicine at Mount Sinai, New York, NY
2. Icahn Institute for Genomics and Multiscale Biology, Icahn School of Medicine at Mount Sinai, New York, NY
3. Department of Surgery, Division of Surgical Oncology, Icahn School of Medicine at Mount Sinai, New York, NY
4. Department of Pathology, Icahn School of Medicine at Mount Sinai, New York, NY
5. Sema4, a Mount Sinai venture, Stamford, CT

^†^equal contribution

^*^Correspondence:

Spiros P Hiotis, MD, PhD

Associate Professor

Department of Surgery

Icahn School of Medicine at Mount Sinai

1470 Madison Avenue, New York, NY, 10029

[spiros.hiotis@mssm.edu](mailto:spiros.hiotis@mssm.edu)

Telephone: 212-241-2891

Jun Zhu, PhD

Professor

Department of Genetics and Genomic Sciences

Icahn Institute of Genomics and Multiscale Biology

Icahn School of Medicine at Mount Sinai

1425 Madison Avenue, New York, NY, 10029

[jun.zhu@mssm.edu](mailto:jun.zhu@mssm.edu)

Telephone: 212-659-8942

**Supplementary Results**

**Prognostic analysis based on gene expression profiles**

In our previous study, we reported differentially expressed genes in non-neoplastic liver between patients of low and high Ishak staged fibrosis (1). The upregulated genes in patients of the high liver fibrosis were significantly enriched for the biological processes cell activation (p-value=1.5×10^-11^), regulation of immune response (p-value=1.7×10^-11^), cell adhesions (p-value=8×10^-10^), and more (listed in Table S14). While much smaller number of genes was upregulated in low liver fibrosis group, those genes were significantly enriched for several biological processes critical to liver function such as organic acid catabolic process (p-value=3.7×10^-19^), carboxylic acid metabolic process (p-value=1.3×10^-16^), oxidation-reduction process (p-value=2.6×10^-8^), and more (Table S14). In addition, these signatures were significantly overlapped with other liver cancer signatures curated in the Molecular Signatures Databases (MsigDB) (2) (Table S2). However, these genes are not associated with tumor recurrence (Figure S2A).

To determine whether gene expression in non-neoplastic liver tissues contains prognostic information, we used 186 survival gene signatures reported by Hoshida *et al*. (3, 4) to classify our samples into good or bad prognostic groups. However, even in non-neoplastic liver tissues the signature genes failed to classified samples into groups with and without tumor recurrence (Figure S2B). The reason for this may be because all of our samples were from HBV infected HCC patients while the signatures were pulled from more heterogeneous HCC patients.

**Examination of HBV integration sites not identified by our pipeline**

There were 7 HBV integration sites reported by Sung et al. not detected by our pipeline. Except for one integration site of sample 65T whose WGS information is not available, we investigated all 6 missed integration sites (chr16:32720350 of 82N; chr2:215692960, chr4:189028263, and chrX:118015438 of 11T; and chr18:3108768 and chr20:11962578 of 200T) based on their raw sequences. In order to find the read support the integration sites, we extracted all reads not aligned and then the reads are chopped into 20 bps. The chopped reads are aligned to HBV genome. If they are fully aligned, we traced back the original reads having the fully aligned chopped reads. Then the remaining region of the original reads were further aligned with the missed sequence integration sites of human sides with flanking regions of 5000 bps. In this way, we could pick individual reads that are partially aligned both to human and HBV as below. However, all these reads contains unaligned regions for both human and HBV explaining the reason our pipeline excluded them as a putative integration site (Figure S3).

**Comparison of HBV integration sites of TCGA HCC-HBV**

Recently, TCGA reported 79 and 92 HBV integration sites from 7 adjacent normal and 37 tumor samples with positive evidence of HBV DNA integration (5). HBV integration sites are identified by two different pipelines performed by BC Cancer Agency and Broad Institute. Our results covered 6 adjacent normal and 5 tumor samples and TCGA reported HBV integration sites are highly consistent with our results (Table S4). Moreover, there are 96 and 12 unique HBV integration sites detected by our method in adjacent normal and tumor tissues while most of TCGA only detected HBV integration sites (47 and 11 in adjacent normal and tumor) are detected only by Broad institute where used simple alignment via TopHat. These results further suggest high sensitivity and selectivity of our method to identify HBV integration sites.

**Functional analysis of host genes with HBV integrations**

**1) Function of host genes with HBV insertion**

To investigate the biological functions of host genes involved HBV fusion transcripts, we performed enrichment analysis of the host genes against GO biological processes. The human genes with HBV fusion transcripts detected in non-neoplastic liver tissues were enriched for cell adhesion (p-value=0.0002), cell junction organization (p-value=0.001), platelet degranulation (p-value=0.002), regulation of phosphate metabolic process (p-value=0.005), and regulation of canonical Wnt receptor signaling pathway (p-value=0.005) (Table S8). Wnt signaling is known to activate β-catenin pathway and promote malignancy in HBV related HCCs (6, 7). In tumors, HBV host genes were enriched for platelet degranulation (p-value=4.9×10^-5^), platelet activation (p-value=9.2×10^-5^), regulation of catalytic activity (p-value=0.001), and negative regulation of signal transduction (p=0.002) (Table S8). Platelets can shelter several viruses including HBV and is known to facilitate their infection at distal sites throughout the circulation (8, 9).

For additional pathway analysis, we also looked up molecular signatures database (MsigDB) and focused on C2, which is a collection of curated gene sets representing expression signatures of genetic and chemical perturbation (2). Only liver-related gene sets were selected and their significance of overlap with host gene was measured. At FET p-value<0.001, only three gene sets (Table S15) overlapped with the HBV fusion genes in non-neoplastic liver tissues. These include up- and down-regulated genes from P53 regulator *MDM4* knockout (10) and down-regulated genes at early fetal liver stage are related with Wnt/β-catenin signaling pathway (11). There was one gene set significantly overlapping with host genes in tumors (Table S15).

**2) Alteration of gene expression by HBV integration**

Whether HBV-human gene fusion events alter the host gene expression was examined. *KMT2B* expression level was higher in tumor tissues with HBV-KMT2B fusion transcripts detected (Figure S7A). *ARAP2* expression levels were different between fusion positive and negative samples (Figure S7B). Several other genes showed different expression patterns but the statistical significance was not observed due to a small sample size.

HBV-KMT2B fusion transcript was detected in four tumor samples, representing the most common transcript in tumor with HBV integration in this cohort (4/21 = 19%). To investigate the potential functional impact of HBV-KMT2B fusion, tumor tissues were separated into HBV-KMT2B fusion transcript positive and negative groups, and gene expression between two groups was compared. Interestingly, HBV-KMT2B fusion positive tumors showed distinct molecular patterns from tumors without HBV integration into *KMT2B* (Figure S7C). A total of 171 differentially expressed genes were identified (fold change>2 & p-value<0.01). Among them, 139 genes were upregulated and 32 were downregulated in HBV-KMT2B fusion positive tumors. The upregulated genes were enriched for several biological processes including complement activation (p-value=6.1×10^-11^), humoral immune response (p-value=3.0×10^-9^), organic acid metabolic process (p-value=7.0×10^-8^), acute inflammatory response (1.9×10^-7^), and other immune response related functions (Table S16). On the other hand, the 32 downregulated genes were enriched for cellular response to growth factor stimulus (p-value = 0.0003), regulation of transforming growth factor beta receptor signaling pathways (p-value =0.004) (Table S16). These results suggest that HBV-KMT2B fusion not only increases *KMT2B* expression but also alters the expression other genes, especially those implicated in immune related functions in tumor.

**Comparison of pathogenic SNPs and somatic mutations with HBV viral loads**

We compared the number of potential pathogenic SNPs and somatic mutations with cccDNA count and HBV replicative activity as we did for the number of host transcripts with HBV integration (Figure S9). No significant association was observed between groups with low and high cccDNA per hepatocyte (Figure S9A and S9C). For HBV replicative activity, the number of potential pathogenic SNPs in non-neoplastic liver tissues significantly decreased in the group with high HBV replicative activity (p<0.05, Figure S9B). We observed similar patterns in tumors, but the association was not statistically significant.

**Microholomog analysis**

Microhomologous sequence between the human and HPV genomes is significantly enriched near integration breakpoints (12), suggesting that MH-mediated DNA repair may be a main mechanism mediating the virus genome integration process. Similar MH sequence enrichment is observed for HBV integrations (12-14).

We followed the analysis method in HBV integration (12, 14) and selected the 5bp-flanking region as in (14). The genome sequences of human and HBV around integration sites were extracted and compared. A MH is defined as the contiguous identical bases in a flaking region. For each integration site, we collected all MHs of different sizes in the flanking region. Finally, we summarized integrations for each MH size. Following (12), we generated the background of MH numbers of random integrations by keeping the HBV integration site and random select loci on human genome as random HBV integration sites on human genome (Figure S10A). Chi-squared test was used to compare the observed and expected numbers of HBV integrations for each MH size.

To the best of our knowledge, this is the first RNAseq-based study that replicated the observation in (13, 14) .

**Categorizing RNAseq reads into genomic locations**

All reads were aligned into human reference genome (hg19) using SpliceMap (15), then each read with available coordinate was further classified into promoter, exon, intron, 3’ UTR, or intergenic with respect to the reference transcriptome.

We also investigated whether HBV integration sites were enriched for specific genomic locations such as CpG island or genomic common or rare fragile sites. The coordinates of CpG islands were downloaded from UCSC genome browser (<http://genome.ucsc.edu>). Then, CpG shores were additionally defined by 2000 bp away from the islands and CpG shelves were 2000 bp away from the shores. For genomic fragile sites, we used 89 common and 29 rare fragile sites reported by Debacker et al (16). Background was defined by (# of reads in genomic regions)/(# of total reads). Chi-square test was performed to test statistical significance and no significance dependency on genomic regions was observed (Figure S10B and S10C).

**Abundance of HBV transcripts**

Gene expression profiling in these 21 pairs of HCC tumor and distant non-neoplastic liver tissue were performed using paired-end (PE) sequencing. To obtain HBV transcript expression, sequence reads were first mapped onto hg19 reference transcript for human genes and unmapped read were further aligned into the HBV reference genome which includes seven Coding Sequences (CDS) corresponding to 4 genes; (1) HBVgp1 – Polymerase, (2) HBVgp2 – S, middle S, and large S protein, (3) HBVgp3 – X protein, (4) HBVgp4 - precore/core and Core and e antigen. The expression level of each gene was measured in terms of Reads Per Kilobase per Million mapped reads (RPKM). The pattern of viral transcript expression across 21 samples is shown in Figure S11. No reads was aligned to S proteins because the CDS of S proteins completely overlapped with the CDS of Polymerase. Other viral genes were widely expressed across samples. We also treated the whole HBV as a single transcript counted the number of reads aligned to HBV as a single gene which is shown as “HBV_all”. There were 4 samples in which no viral transcript was detected; P99 and P106 in tumors P106, P161 in non-neoplastic liver samples which were consistent with their low level of HBV DNA in their hepatocytes.

The expression of HBVgp1_Polymerase marginally correlated with Ishak fibrosis stage (Spearman correlation p-value <0.05 for both tumor and non-neoplastic liver tissues). The HBV gene expression associated with HBV DNA in both tumor and non-neoplastic liver tissues (Spearman p-value <0.01 and <0.02 correspondingly). Expression of HBV genes did not differ between tumor and paired non-neoplastic liver in all three genes (t-test p-value= 0.97, 0.68, 0.35, 0.70 for HBV_all, HBVgp1_P, HBVgp3_X, and HBVgp4_PreC, respectively). This group of patients has small tumors (<5cm). The differences in HBV DNA and cccDNA between tumor and non-neoplastic liver are only significant in large tumors when analyzed based on cutoff of 3cm.

**Supplementary Methods**

**A robust pipeline for identifying HBV integration sites**

**1) Improvement from original VirusFinder**

First, in the data preprocessing step, VirusFinder uses Bowtie2 (17) to align the sequence reads to human genome, then the unmapped reads are collected for detecting viral species and virus-integration site. We set more stringent criteria for local alignments to human genome and generated a larger pool of candidate reads for mapping to virus genome. Second, in the integration detection step, VirusFinder uses BWA-ALN (18) to align the reads collected from the data preprocessing step to the new reference genomes including both human genome and the detected virus genome. Soft-clipped reads from the alignment provide vital information to detect the exact integration loci. In our modified pipeline, we used BWA-MEM (19) to leverage longer sequence reads, which were not well handled by BWA-ALN. It has been shown that soft-clipped reads, which become more prevail with longer read length, are better handled by BWA-MEM (19). Given that soft-clipped reads are one of the major sources to precisely detect integration sites, our pipeline is more sensitive and accurate in detecting virus integration sites for both WGS and RNAseq dataset (Figure 3C and 3D). Third, SVdetect (20) is used to detect the potential region containing integration sites based on discordant paired-end reads. CREST (21) is used to further refine these potential regions based on soft-clipped reads. In VirusFinder, reads passed CREST filtering are reported as putative integration sites and assigned as high confidence. Candidate integrations failed to pass CREST filtering procedure are reported as low confidence putative integration sites. For the case that there is no output from CREST on the potential region detected by SVdetect, the loci in the region are sorted in descending order of the number of soft-clipped reads. The first two ranked loci are also reported as low confidence putative integration sites. However, after checking the soft clip reads reported by VirusFinder in detail, we found that most these soft-clipped reads are not always mapped to HBV genome by BWA-MEM. On the other hand, there are a lot of inconsistencies between discordant paired-end and soft-clipped reads. For example, the location of soft-clipped part is not mapped to human genome by BWA-MEM or even the soft-clipped reads are mapped to human chromosomes different from the one based on discordant paired-end reads. In our pipeline, we refined low confidence integration loci reported by CREST and re-mapped the soft-clipped parts to the HBV genome with BLAT (-tileSize=7 -stepSize=1 -out=psl -minScore=15 -noHead -maxIntron=1). Last, there are no explicit thresholds for discordant paired-end and soft-clipped reads to define an integration site. In our pipeline we imposed thresholds on numbers of supporting reads for discordant paired-end and soft-clipped reads as 3 and 1, respectively, which were tuned according to simulation studies (Figure S12).

**2) Simulation studies**

*Simulation of RNAseq data*

In order to select the optimal threshold on soft clipped reads and discordant reads pair, we implemented large-scale simulations of RNAseq data. Known transcripts, Homo_sapiens.GRCh37.75.cdna.all.fa, were downloaded from ensemble database (<http://useast.ensembl.org/info/data/ftp/index.html?redirect=no>). There are 7375 known transcripts on Chr5. The relative gene expression difference was incorporated into the simulation by using the RPKM information from Chr5 of sample P105D in the Mount Sinai dataset. Due to the RPKM is generated based on transcript definition of reference transcript of hg19 from UCSC, we chose the best matched transcript from ensemble database for each gene. 463 transcripts are collected with corresponding relative coverage ratio calculated based on their RPKM and length. Given designed coverage, the specific coverage on each transcript was selected as [designed coverage * relative coverage ratio].

In each round of simulation, a hundred of them were selected to simulate HBV integrated transcripts, on which an integration site was randomly assigned. The integrated HBV sequence was randomly selected across the HBV genome (The NCBI HBV genome version NC_003977.1 (length=3215bp)). For the purpose of mimicking the heterogeneous frequency of HBV integrated transcripts, the reads on each constructed HBV integrated transcript were generated according to a predefined insertion allele frequency. For example, assuming the coverage on a transcript is *C* based on designed coverage and relative coverage ratio*,* if an insertion allele frequency *x* (scale 0 to 1) is assigned to a HBV integrated transcript, then coverage of reads on this HBV integrated transcript is *x***C*, while coverage of reads generated on the corresponding normal transcript used to construct the HBV integrate transcript is (1-*x*)**C*. The number of transcript for each frequency is described in Table S17.

| **Frequency** | **1%** | **2%** | **5%** | **10%** | **25%** | **50%** | **100%** |
| --- | --- | --- | --- | --- | --- | --- | --- |
| Number of transcript | 20 | 20 | 15 | 15 | 10 | 10 | 10 |

**STable 17**. The number of transcripts of different HBV integration frequency.

The designed coverage was selected as 10, 20, 30, 50, 100 and 200. The coverage *C* on each transcript is calculated with relative coverage ratio. The reads for each transcript were generated with pIRS (22). Read length and insert size were selected as 100 and 286 (estimated from P105D with Picard) respectively (23). The simulation was repeated for 100 times. In each round, the generated reads were analyzed by our pipeline or the original VirusFinder, and then the predicted integration sites were mapped back to hg19.

The performance is evaluated based on resolution of 500bp. If the distance between a predicted integration site and the simulated integration site is less than the resolution, the predicted integration site is accepted as a true positive. The recall increases significantly with coverage while the precision does not change significantly with different coverage (Figure S12). Lower coverage even has better precision than high coverage which validates that our control on the soft clipped reads and discordant pairs is especially necessary for high sequencing coverage.

The F-score was the best with number of soft-clipped read>=1 and number of discordant pairs >=2 for coverage 200 (Figure S12C). Considering that we prefer high precision integration prediction in this study, we choose to increase the threshold of discordant pair to 3. In such a case, we will have better precision and similar recall to the optimized case.

We compared with VirusFinder using the simulated data based on F Score. The parameters for the modified pipeline were set as soft-clipped reads>=1 & discordant pairs >=3. Comparison shows that the modified pipeline performs better than VirusFinder under different coverage setting (Figure 3D).

*Simulation of DNA sequencing data*

For WGS sequencing data, we evaluated the performance of our pipeline on integrations across different frequency and different coverage. Similarly to RNAseq, Chr5 (length=180915261bp) was selected as the host genome. For each round of simulation, we assumed there were 100 different alleles (different cells). For 1% insertion frequency, a unique integration site was simulated for each allele. For 2% insertion frequency 2 alleles (cells) were randomly selected, then an integration sites was added to the two alleles. The process was repeated according to the predefined insertion allele (Table S18).

| **IAF** | **1%** | **2%** | **5%** | **10%** | **25%** | **50%** | **100%** |
| --- | --- | --- | --- | --- | --- | --- | --- |
| Number of insertion events | 100 | 50 | 20 | 10 | 4 | 2 | 1 |

**STable 18**. Number of repeats for different HBV insertion allele frequencies in WGS.

The 50% and 100% integration sites were randomly selected from the known ones in Sung et al (13). Other integration sites were uniformly distributed on chr5 except beginning and ending 50k. The length of integration HBV was uniformly selected across HBV genome. pIRS was used to simulate the pair-ended reads for each alleles with coverage of 20, read length of 90 and fragment size of 500 (24). Other parameters were used as default. For a specific coverage, reads were randomly selected from each allele in equal number. The HBV related reads (discordant and soft-clipped reads) and human reads were selected separately to keep the ratio between them the same as in the original simulated reads data for each allele. The simulations were iterated for 100 rounds.

The performance is evaluated with a resolution of 5bp. Similar as the result of RNAseq simulations the precision is not sensitive to coverage (Figure S13A). Recall increases with higher coverage (Figure S13B). According to F Score (Figure S13C), the optimized parameter is selected as soft clipped reads >=1 & discordant pair >=0.

We also compared our pipeline with VirusFinder using the simulated DNA data based on F Score. Our pipeline performed better than VirusFinder under different coverage settings (Figure 3C).

The relationships between True Positive Rate (TPR: number of positive prediction / number of true insertions), Coverage and Insertion Allele Frequency are displayed in Figure 3B. With the fixed allele frequency, the TPR increases with higher coverage. In the same way, TPR increases with higher frequency with a given coverage. This indicates that with the commonly used coverage of WGS we may be potentially missing a large number of integration sites with low frequency.

**Data preprocessing and QC**

**1) Gender match**

Gender information can be inferred from gene expression or other molecular data. *RPS4Y1* (Ribosomal protein S4, Y-linked 1) is highly expressed in male (25). Its expression level can be used to robustly classify samples into male and female (26). For our 42 RNAseq samples (2 X 21 patients), we inferred gender of each patient based on the expression of RPS4Y1 and confirmed that predicted gender agreed with the gender in clinical information provided in Table S1 (Figure S14A). For BGI samples, gender information is not available.

**2) Matching pairs of tumor and non-neoplastic liver tissues**

While gender information matched well, it is still possible that samples swapp or other errors occur in the process of data preparation. In order to confirm that each non-neoplastic liver tissue is paired with its matching tumor tissues, genotypes of all samples were inferred from RNAseq profiles then used to match between non-neoplastic liver and tumor tissues. While it is not trivial to map transcriptome reads onto the genome due to its unequal transcript expression levels but low resolution still fits for our purpose for sample comparison. We used “mpileup” function in samtools (27) to infer SNPs then filtered out locus with sum of read depths >100 (Figure S14B). The mutational status of selected 35504 loci was compared between a non-neoplastic liver tissue and tumor tissues as depicted in Figure S14C. The matched pairs would show a significantly higher correlation compared to other. For example, the non-neoplastic liver tissue of P158 (P158D) is correlated with its own tumor tissue (P158A) with the highest coefficient compared to other tumor tissues as shown in Figure S14D. Based on the same way, we confirmed that all pairs in Mt. Sinai dataset and 9 pairs in BGI dataset were best-matched with its own pairs.

**Supplementary Figure Legends**

**Figure S11: HBV gene expression in tumor and non-neoplastic liver tissues form 21 patients.** Heatmaps show RPKM values of HBV genes (Polymerase, X protein, and PreCore). HBV_All shows the HBV genome as a single gene. No reads were aligned to S proteins so the panel for the gene is not inlcluded. Ishak liver fibrosis stage and tumor recurrence (0=no and 1=yes) correspodning to each patient is shown.

**Figure S12: Evaluation of prediction accuracy in simulations on RNAseq data.** **A)** Precision (number of accurate prediction/number of all prediction), **B)** Recall (number of accurate prediction/number of real integration), **C)** F-score (2X[(precision*recall)/(precision+recall)]).

**Figure S13: Evaluation of prediction accuracy in simulations on DNAseq data.** **A)** Precision (number of accurate prediction/number of all prediction), **B)** Recall (number of accurate prediction/number of real integration), **C)** F-score (2X[(precision*recall)/(precision+recall)]).

**Figure S14: Preprocessing of RNAseq dataset. A) Gender consistency.** Gender of 21 patients is predicted by the expression of *RPS4Y1* expression for both tumor and non-neoplastic liver tissues. With the threshold of 2, *RPS4Y1* expression can be separated into two groups clearly. All predicted genders agree well with genders provided in clinical information. **B)** Only loci with high read depth has been used for the comparison. **C)** Each normal tissue is matched with all tumor tissue based on their genotypes. **D)** The best correlation is expected when they are matched pairs. In this example, the genotype of P158D and P158A is significantly higher correlated than any other samples.

**REFERENCES**

1. Wang Q, Lin L, Yoo S, Wang W, Blank S, Fiel MI, Kadri H, et al. Impact of non-neoplastic vs intratumoural hepatitis B viral DNA and replication on hepatocellular carcinoma recurrence. Br J Cancer 2016;115:841-847.

2. Subramanian A, Tamayo P, Mootha VK, Mukherjee S, Ebert BL, Gillette MA, Paulovich A, et al. Gene set enrichment analysis: a knowledge-based approach for interpreting genome-wide expression profiles. Proc Natl Acad Sci U S A 2005;102:15545-15550.

3. Hoshida Y, Villanueva A, Kobayashi M, Peix J, Chiang DY, Camargo A, Gupta S, et al. Gene expression in fixed tissues and outcome in hepatocellular carcinoma. N Engl J Med 2008;359:1995-2004.

4. Hoshida Y, Villanueva A, Sangiovanni A, Sole M, Hur C, Andersson KL, Chung RT, et al. Prognostic gene expression signature for patients with hepatitis C-related early-stage cirrhosis. Gastroenterology 2013;144:1024-1030.

5. Cancer Genome Atlas Research Network. Electronic address wbe, Cancer Genome Atlas Research N. Comprehensive and Integrative Genomic Characterization of Hepatocellular Carcinoma. Cell 2017;169:1327-1341 e1323.

6. Merle P, de la Monte S, Kim M, Herrmann M, Tanaka S, Von Dem Bussche A, Kew MC, et al. Functional consequences of frizzled-7 receptor overexpression in human hepatocellular carcinoma. Gastroenterology 2004;127:1110-1122.

7. Merle P, Kim M, Herrmann M, Gupte A, Lefrancois L, Califano S, Trepo C, et al. Oncogenic role of the frizzled-7/beta-catenin pathway in hepatocellular carcinoma. J Hepatol 2005;43:854-862.

8. Assinger A. Platelets and infection - an emerging role of platelets in viral infection. Front Immunol 2014;5:649.

9. Iannacone M, Sitia G, Ruggeri ZM, Guidotti LG. HBV pathogenesis in animal models: recent advances on the role of platelets. J Hepatol 2007;46:719-726.

10. Martoriati A, Doumont G, Alcalay M, Bellefroid E, Pelicci PG, Marine JC. dapk1, encoding an activator of a p19ARF-p53-mediated apoptotic checkpoint, is a transcription target of p53. Oncogene 2005;24:1461-1466.

11. Cairo S, Armengol C, De Reynies A, Wei Y, Thomas E, Renard CA, Goga A, et al. Hepatic stem-like phenotype and interplay of Wnt/beta-catenin and Myc signaling in aggressive childhood liver cancer. Cancer Cell 2008;14:471-484.

12. Hu Z, Zhu D, Wang W, Li W, Jia W, Zeng X, Ding W, et al. Genome-wide profiling of HPV integration in cervical cancer identifies clustered genomic hot spots and a potential microhomology-mediated integration mechanism. Nat Genet 2015;47:158-163.

13. Sung WK, Zheng H, Li S, Chen R, Liu X, Li Y, Lee NP, et al. Genome-wide survey of recurrent HBV integration in hepatocellular carcinoma. Nat Genet 2012;44:765-769.

14. Zhao LH, Liu X, Yan HX, Li WY, Zeng X, Yang Y, Zhao J, et al. Genomic and oncogenic preference of HBV integration in hepatocellular carcinoma. Nat Commun 2016;7:12992.

15. Au KF, Jiang H, Lin L, Xing Y, Wong WH. Detection of splice junctions from paired-end RNA-seq data by SpliceMap. Nucleic Acids Res 2010;38:4570-4578.

16. Debacker K, Kooy RF. Fragile sites and human disease. Hum Mol Genet 2007;16 Spec No. 2:R150-158.

17. Langmead B, Salzberg SL. Fast gapped-read alignment with Bowtie 2. Nat Methods 2012;9:357-359.

18. Li H, Durbin R. Fast and accurate long-read alignment with Burrows-Wheeler transform. Bioinformatics 2010;26:589-595.

19. Li H. Aligning sequence reads, clone sequences and assembly contigs with BWA-MEM. In; 2013.

20. Zeitouni B, Boeva V, Janoueix-Lerosey I, Loeillet S, Legoix-ne P, Nicolas A, Delattre O, et al. SVDetect: a tool to identify genomic structural variations from paired-end and mate-pair sequencing data. Bioinformatics 2010;26:1895-1896.

21. Wang J, Mullighan CG, Easton J, Roberts S, Heatley SL, Ma J, Rusch MC, et al. CREST maps somatic structural variation in cancer genomes with base-pair resolution. Nat Methods 2011;8:652-654.

22. Xu J, Yun X, Jiang J, Wei Y, Wu Y, Zhang W, Liu Y, et al. Hepatitis B virus X protein blunts senescence-like growth arrest of human hepatocellular carcinoma by reducing Notch1 cleavage. Hepatology 2010;52:142-154.

23. Kang L, Liu X, Gong Z, Zheng H, Wang J, Li Y, Yang H, et al. Genome-wide identification of RNA editing in hepatocellular carcinoma. Genomics 2015;105:76-82.

24. Hu X, Yuan J, Shi Y, Lu J, Liu B, Li Z, Chen Y, et al. pIRS: Profile-based Illumina pair-end reads simulator. Bioinformatics 2012;28:1533-1535.

25. Karyagyna AS, Vassiliev MO, Ershova AS, Nurtdinov RN, Lossev IS. Probe-Level Universal Search (PLUS) algorithm for gender differentiation in affymetrix datasets. J Bioinform Comput Biol 2010;8:553-577.

26. Yoo S, Huang T, Campbell JD, Lee E, Tu Z, Geraci MW, Powell CA, et al. MODMatcher: multi-omics data matcher for integrative genomic analysis. PLoS Comput Biol 2014;10:e1003790.

27. Ramirez-Gonzalez RH, Bonnal R, Caccamo M, Maclean D. Bio-samtools: Ruby bindings for SAMtools, a library for accessing BAM files containing high-throughput sequence alignments. Source Code Biol Med 2012;7:6.
